# Phosphine-promoted [4 + 3] annulation of allenoate with aziridines for synthesis of tetrahydroazepines: phosphine-dependent [3 + 3] and [4 + 3] pathways†

**DOI:** 10.1039/c8ra09852b

**Published:** 2019-01-09

**Authors:** Honglei Liu, Yan Lin, Yan Zhao, Miaoren Xiao, Leijie Zhou, Qijun Wang, Cheng Zhang, Dongqi Wang, Ohyun Kwon, Hongchao Guo

**Affiliations:** Department of Applied Chemistry, China Agricultural University Beijing 100193 China hchguo@cau.edu.cn; Institute of High Energy Physics, Chinese Academy of Science 19B Yuquan Lu, Shijingshan District Beijing 100049 P. R. China; Department of Chemistry and Biochemistry, University of California Los Angeles California 90095-1569 USA ohyun@chem.ucla.edu

## Abstract

In this manuscript, phosphine-dependent [3 + 3] and [4 + 3] annulation reactions of allenoate with aziridines were disclosed. The alkyldiphenylphosphine-promoted [4 + 3] annulation of allenoate with aziridines has been achieved under mild conditions, providing biologically interesting functionalized tetrahydroazepines in moderate to excellent yield with moderate to excellent regioselectivity and diastereoselectivity.

## Abstract 2

Phosphine-dependent [3 + 3] and [4 + 3] annulation reactions of allenoate with aziridines were disclosed. The alkyldiphenylphosphine-promoted [4 + 3] annulation produced tetrahydroazepines.

## Graphical abstract

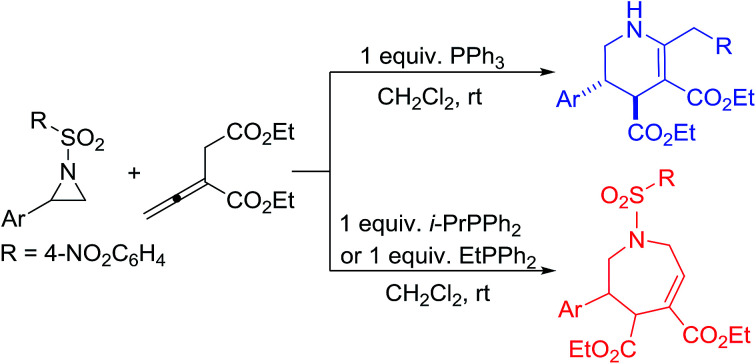

Nitrogen-containing heterocyclic compounds are widely present in biologically active natural products and synthetic pharmaceuticals. Among them, tetrahydropyridines which can be converted into pyridines and piperidines are intriguing synthetic targets due to their significant biological activities.^1^ In addition, azepines are widely found as the core structure in a large number of compounds that possess important pharmaceutical activities. The compounds containing the azepine moiety are important targets in synthetic and medicinal chemistry.^2^ Among these compounds (Fig. 1), azelastine is an effective and safe treatment agent for urticaria.^3^ Meptazinol is a new opioid-type analgesic with mixed agonist/antagonist properties.^4^ (−)-Balanol is a fungal metabolite with potent protein kinase C inhibitory properties.^5^ An anticonvulsant, carbamazepine, is known to show incidences of cutaneous adverse drug reactions including Stevens–Johnson syndrome, toxic epidermal necrolysis and drug-induced hypersensitivity syndrome.^6^ Epinastine is a potent antiallergic agent that not only has antihistaminic property but also provides antileukotriene, anti-PAF and antibradykinin activities.^7^ The tetracyclic natural product, (−)-tetrapetalone A is a novel lipoxygenase inhibitor from *Streptomyces* sp.^8^ Therefore, new synthetic methodologies for the synthesis of azepine derivatives have attracted much attention. Among various methods, the cycloaddition reactions are practical and efficient methods, and have been extensively investigated.

**Fig. 1:**
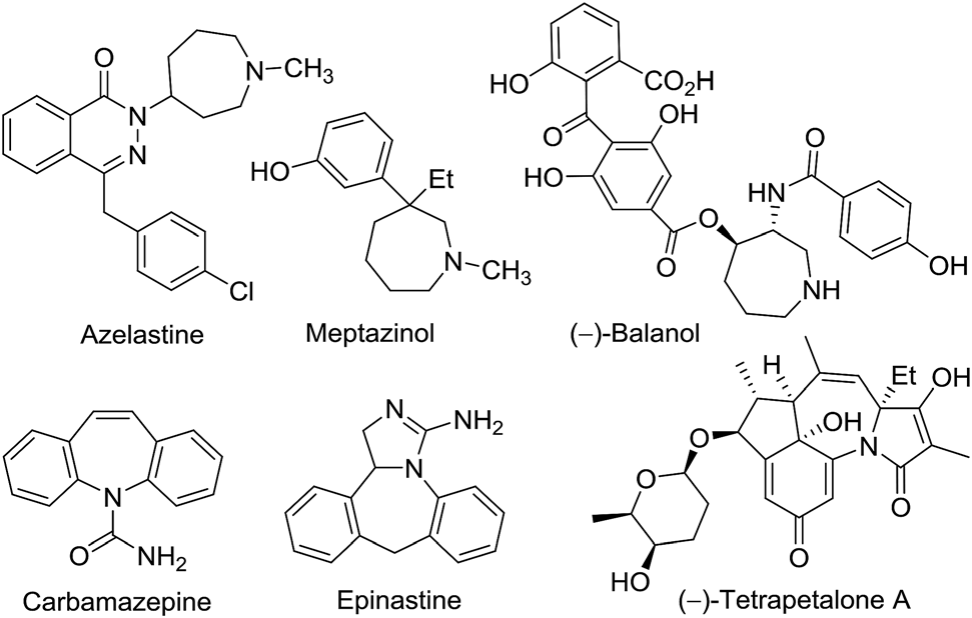
Selected examples of biologically active azepine-containing heterocyclic compounds.

Nucleophilic phosphine-catalyzed cycloaddition reactions of allenoates have evolved as a very useful tool to access various complex ring systems of organic molecules.^9,10^ Since Lu and coworkers reported the first phosphine-catalyzed [3 + 2] cycloaddition of allenoates with electron-deficient alkenes in 1995,^11^ various types of cycloaddition reactions have been developed to afford different sizes of carbocycles or heterocycles.^9^ In spite of these advances, developing new cycloaddition reaction of allenoates is still of great significance to construct novel ring frameworks with functional groups.

Aziridines are an important type of versatile building blocks for synthesis of diverse nitrogen-containing heterocyclic compounds and natural products.^12^ In the presence of Lewis acid or organocatalyst, aziridines may undergo a ring-opening reaction through C–N bond cleavage and work as a masked 1,3-dipole to react with various dipolarophiles, giving diverse cycloadducts. Many Lewis acid or organocatalyst-mediated cycloaddition reactions such as [3 + 2],^13^ [3 + 3],^14^ [6 + 3]^15^ and [8 + 3]^16^ cycloaddition reactions involving aziridines have been reported. In 2009, Kwon reported the first PPh_3_-promoted [3 + 3] annulation of aziridines with α-substituted allenoates to generate highly functionalized tetrahydropyridines by release of SO_2_.^17*a*^ During the process, aziridines undergo a ring-opening reaction through the breakage of the C–N bond upon the attack of the zwitterionic adduct formed by the addition of PPh_3_ to an allenoate, and the resulting amide anion attacks the β-carbon of the allenoate after an intramolecular desulfonation to realize the [3 + 3] annulation (Scheme 1).^17^ The reaction is operationally simple and produces highly functionalized tetrahydropyridines in good to excellent yields with high levels of diastereoselectivity. In theory, however, the amide anion without the desulonation could attack the γ-carbon of the allenoates to result in a [4 + 3] annulation (Scheme 1).^18^ With this query in mind and our continuing interest in phosphine-catalyzed cycloaddition reactions,^19^ we herein report the first alkyldiphenylphosphine-promoted [4 + 3] annulation of aziridines with an allenoate to afford functionalized tetrahydroazepines under simple and mild reaction conditions (Scheme 1).

**Scheme 1:**
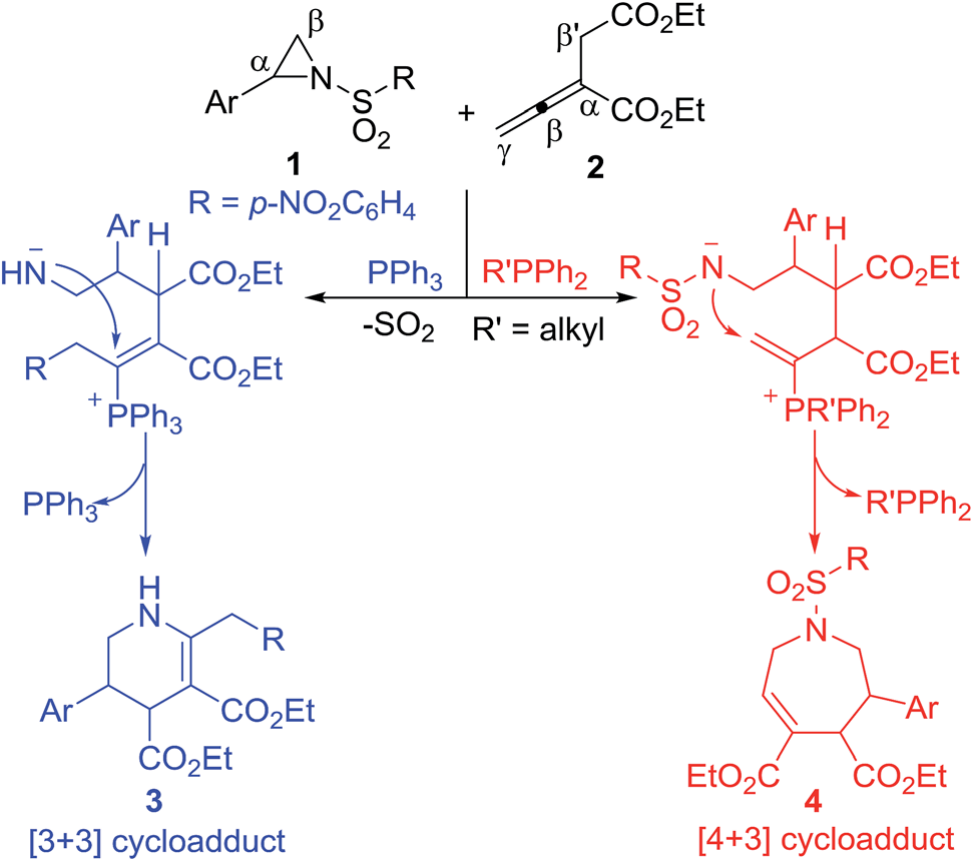
Phosphine-dependent [3 + 3] and [4 + 3] annulation of allenoate with aziridines.

As shown in Scheme 1, in our previous work, in the presence of Ph_3_P, aziridines and α-substituted allenoates performed [3 + 3] annulation in dichloromethane at room temperature. Through revisiting the catalyst screening, we found that alkyldiphenyl-phosphines can reverse the regioselectivity, leading to [4 + 3] annulation, as shown in Table 1. The best result for [4 + 3] annulation of aziridine 1a and allenoate 2 was obtained when 1 equivalent of EtPPh_2_ was added, with 93% yield of the cycloadducts, 92 : 8 of regioselectivity and 81 : 19 of diastereoselectivity (Table 1, entry 3). *n*-PrPPh_2_ is also an effective catalyst compared to PPh_3_, and gave similar result to that with EtPPh_2_ (entry 4). MePPh_2_, i-PrPPh_2_, *n*-BuPPh_2_, CyPPh_2_, DPPB, and DPPP gave good yield of cycloadducts with poor to moderate regioselectivity (entries 2, 5, 6, 8–11). *t*-BuPPh_2_ afforded much lower yield of cycloadducts although with excellent regio- and diastereoselectivity (100 : 0) (entry 7). Subsequently, the effect of solvents was evaluated with the model reaction using EtPPh_2_ as the catalyst. The results showed that the aprotic CH_2_Cl_2_ remained to be the best solvent, while MeOH gave excellent reaction selectivity but low yield of cycloadducts (entry 16). Other solvents, such as THF, CH_3_Cl, Cl(CH_2_)_2_Cl, and toluene afforded low to moderate yield of cyloadducts and lower reaction selectivity (entries 12–15). As such, CH_2_Cl_2_ was selected as the best solvent for the reaction. The relative configuration of the product 4a was determined by single-crystal X-ray analysis.^20^

**Table 1:** Screening of the reaction conditions^a^

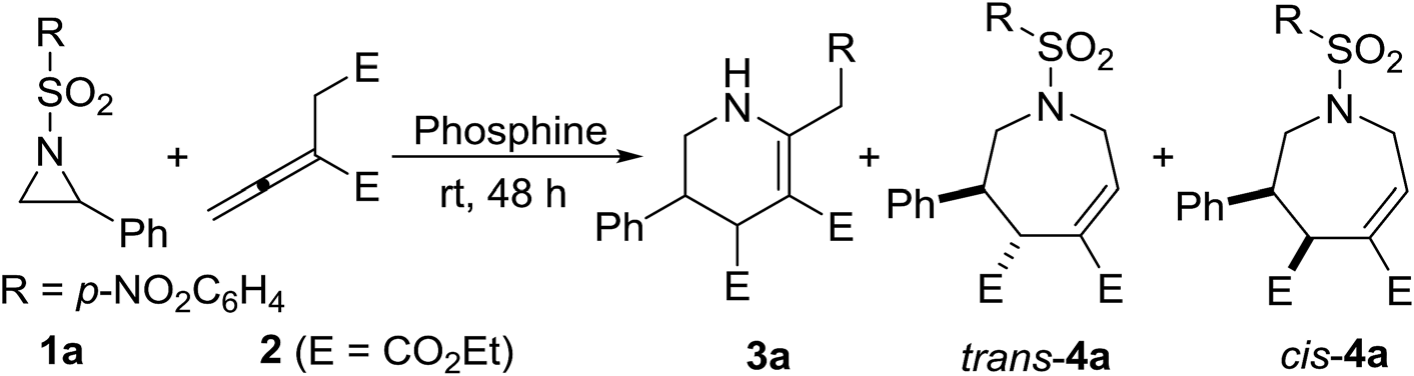					
Entry	Phosphine (mol%)	Solvent	Yield^b^ (%)	4a : 3a^c^	dr (*trans* : *cis*) for 4a^c^
1	PPh_3_ (100)	CH_2_Cl_2_	73	0 : 100	—
2	MePPh_2_ (100)	CH_2_Cl_2_	78	90 : 10	54 : 46
3	EtPPh_2_ (100)	CH_2_Cl_2_	93	92 : 8	81 : 19
4	*n*-PrPPh_2_ (100)	CH_2_Cl_2_	97	80 : 20	91 : 1
5	i-PrPPh_2_ (100)	CH_2_Cl_2_	35	63 : 37	100 : 0
6	*n*-BuPPh_2_ (100)	CH_2_Cl_2_	56	89 : 11	78 : 22
7	*t*-BuPPh_2_ (100)	CH_2_Cl_2_	21	100 : 0	100 : 0
8	CyPPh_2_ (100)	CH_2_Cl_2_	83	60 : 40	82 : 18
9	DPPB (100)	CH_2_Cl_2_	35	66 : 34	100 : 0
10	DPPB (50)	CH_2_Cl_2_	57	77 : 23	100 : 0
11	DPPP (50)	CH_2_Cl_2_	48	69 : 31	100 : 0
12	EtPPh_2_ (100)	Cl(CH_2_)_2_Cl	43	70 : 30	30 : 70
13	EtPPh_2_ (100)	CHCl_3_	44	73 : 27	62 : 38
14^d^	EtPPh_2_ (100)	Toluene	42	60 : 40	84 : 16
15^d^	EtPPh_2_ (100)	THF	66	85 : 15	80 : 20
16^d^	EtPPh_2_ (100)	MeOH	32	100 : 0	100 : 0

a Unless otherwise stated, all reactions were performed using 0.125 mmol of 1a and 0.150 mmol of 2 in 5 mL of CH_2_Cl_2_ at room temperature for 48 h.

b Sum of the isolated yields of 3a and 4a.

c Ratio of isolated yields.

d React time is 72 h. DPPB: 1,4-bis(diphenylphosphino)butane; DPPP: 1,3-bis(diphenylphosphino)propane.

Under the optimized conditions, the annulation reactions of different aryl substituted aziridines with diethyl 2-vinylidenesuccinate were evaluated (Table 2). In most cases, regardless of the electronic nature of the substituent of the aryl group, using EtPPh_2_ or *n*-PrPPh_2_ as the catalyst, moderate to good yield and moderate to good selectivity of cycloadducts were obtained, and the yields are usually lower than that having the simple phenyl ring. The position of substituents on the benzene ring seems to have no significant influence on reactivity and selectivity. For example, substituents such as 4-MeC_6_H_4_ and 2,4,6-Me_3_C_6_H_2_ gave the desired products 4d and 4g in similar yields (entries 4 and 7). The annulation reaction also worked well with 2-naphthyl substituted aziridine (1n), affording the corresponding product in 58% yield (entry 14). Unfortunately, the alkyl substituent gave no desired product, due to the weak electrophilic properties of alkyl aziridines. All these products (4) are new compounds.

**Table 2:** Substrate scope with respect to aziridines^a^

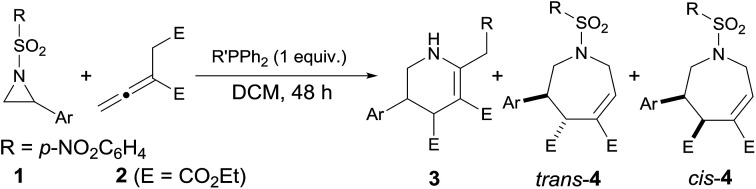							
Entry	Ar in 1	R′PPh_2_	*T*/°C	Yield^b^ (%) of 4 + 3	4 : 3^c^	4	dr (*trans* : *cis*) for 4^c^
1	C_6_H_5_, 1a	EtPPh_2_	25	93	92 : 8	4a	81 : 19
2	2-MeC_6_H_4_, 1b	*n*-PrPPh_2_	25	65	66 : 34	4b	84 : 16
3	3-MeC_6_H_4_, 1c	*n*-PrPPh_2_	25	58	79 : 21	4c	71 : 29
4	4-MeC_6_H_4_, 1d	*n*-PrPPh_2_	20	72	88 : 12	4d	86 : 14
5	2,4-Me_2_C_6_H_3_, 1e	EtPPh_2_	25	96	92 : 8	4e	61 : 39
6	2,5-Me_2_C_6_H_3_, 1f	*n*-PrPPh_2_	20	46	93 : 7	4f	81 : 19
7	2,4,6-Me_3_C_6_H_2_, 1g	*n*-PrPPh_2_	25	77	82 : 18	4g	62 : 38
8	4-*t*-BuC_6_H_4_, 1h	*n*-PrPPh_2_	25	57	84 : 16	4h	78 : 22
9	2-FC_6_H_4_, 1i	*n*-PrPPh_2_	25	60	63 : 37	4i	75 : 25
10	3-FC_6_H_4_, 1j	*n*-PrPPh_2_	25	48	75 : 25	4j	88 : 12
11	4-FC_6_H_4_, 1k	*n*-PrPPh_2_	20	73	73 : 27	4k	70 : 30
12	2-ClC_6_H_4_, 1l	*n*-PrPPh_2_	25	78	77 : 23	4l	80 : 20
13	2-BrC_6_H_4_, 1m	*n*-PrPPh_2_	20	42	60 : 40	4m	72 : 28
14	2-Naphthyl, 1n	*n*-PrPPh_2_	25	58	81 : 19	4n	78 : 22

a All of the reactions were performed using 0.125 mmol of 1a, 0.150 mmol of 2, and 0.125 mmol of catalyst in 5 mL of CH_2_Cl_2_ for 48 h.

b Sum of the isolated yields of 3 and 4.

c Ratio of isolated yields.

Two plausible pathways for the reactions of the aziridines 1 and the allenoate 2 are presented in Scheme 2. PPh_3_ and EtPPh_2_ or *n*-PrPPh_2_ were found to mainly lead to [3 + 3] and [4 + 3] annulations, respectively. The reaction starts with a nucleophilic addition of the catalyst to the allenoate 2. A subsequent proton transfer then occurs to neutralize the negative charge on the terminal γ-carbon atom of 5. The newly formed secondary carboanion 6 is nucleophilic, and may attack the electron-deficient C atom of the aziridine to give a zwitterionic intermediate 7. When PPh_3_ is used as catalyst, a proton transfer ensues to neutralize the negative charge on N atom and results in a primary carboanion 8. The formation of 8 may be followed by a desulfonylation step and the *p*-nitrophenyl group is migrated to the terminal γ-carbon, releasing a molecule of SO_2_ and leaving the negative charge on the N atom. A nucleophilic step then occurs to close the six-membered ring and the elimination of triphenylphosphine gives the [3 + 3] annulation product 3 with the catalyst being regenerated. Compared with PPh_3_, when alkyldiphenylphosphine is used as catalyst, the primary carboanion 11 isomerizes into intermediate 12, which performs a proton transfer from N atom to C atom to give the intermediate 13. The cyclization of 13 furnished the ylide 14, which undergoes a proton transfer to produce the intermediate 15. Through elimination of the phosphine, the β-phosphonium ester 15 was converted to the [4 + 3] annulation product 4. The carbon–carbon single bond between C_4_ and C_5_ in the intermediates 11, 12 and 13 might rotate, thus resulting in moderate diastereoselectivity.

**Scheme 2:**
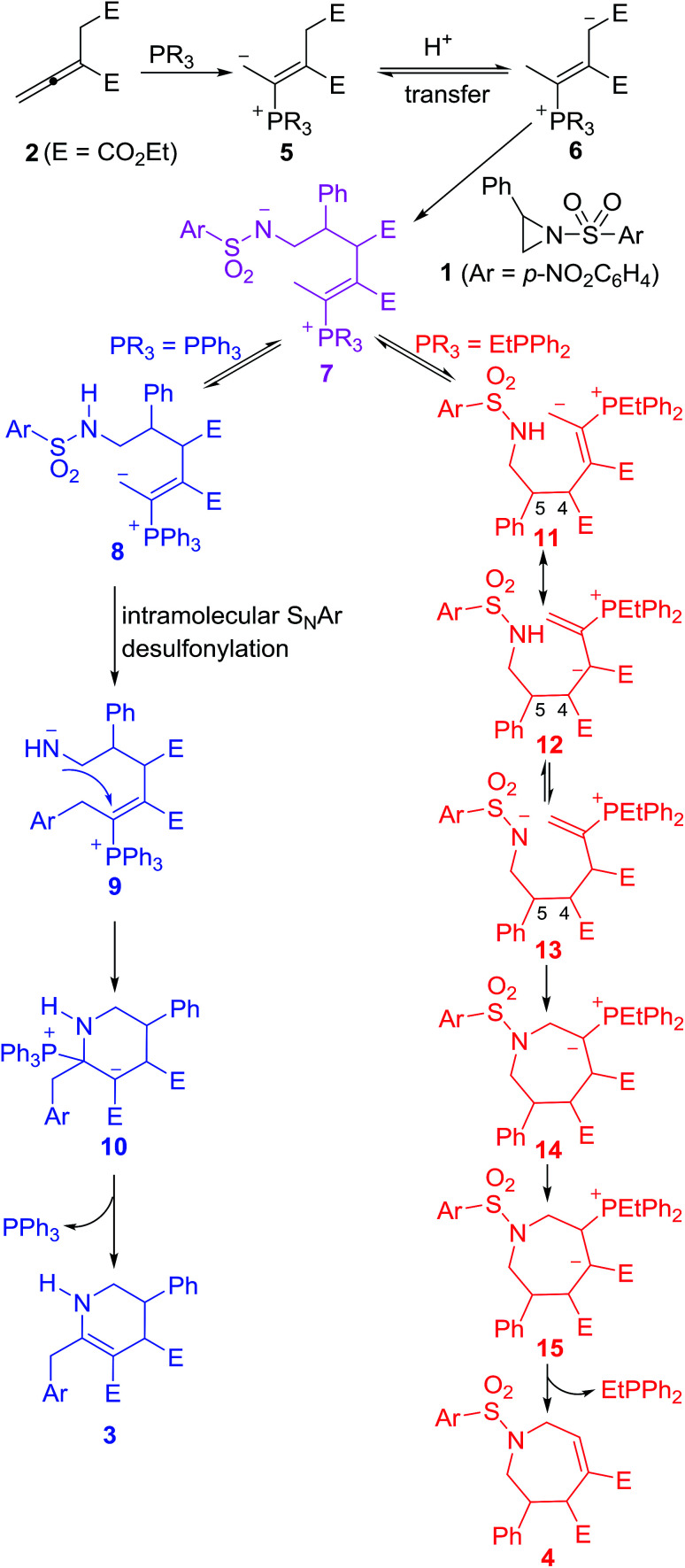
The stepwise pathways of the [3 + 3] and [4 + 3] annulation reactions.

## Conclusions

In conclusion, we disclosed phosphine-dependent [3 + 3] and [4 + 3] annulations of allenoate with aziridines and developed the first phosphine-promoted [4 + 3] annulation involving aziridines. The reaction works efficiently under mild conditions to give functionalized tetrahydroazepines in moderate to excellent yield with moderate to excellent diastereoselectivity.

## Experimental

### General methods

All reactions were performed under N_2_ atmospheres in oven-dried glassware with magnetic stirring. Unless otherwise stated, all reagents were purchased from commercial suppliers and used without further purification. All solvents were purified and dried according to standard methods prior to use. Organic solutions were concentrated under reduced pressure on a rotary evaporator or an oil pump. Reactions were monitored through thin layer chromatography (TLC) on silica gel-precoated glass plates (0.25 mm thickness, silica gel). Chromatograms were visualized by fluorescence quenching with UV light at 254 nm. Flash column chromatography was performed using flash silica gel (200–300 mesh). ^1^H and ^13^C NMR spectra were recorded in CDCl_3_ using a 300 MHz NMR instrument (referenced internally to Me_4_Si). Data for ^13^C NMR spectra are reported in terms of chemical shift. Melting points were determined on a melting point apparatus.

### Preparation of aziridines 1

The 2-aryl-1-(4-nitrobenzenesulfonyl) aziridines were prepared according to procedures described previously in the literature.^17*a*^

### Preparation of allenoate 2

The diethyl 2-vinylidenesuccinate 2 was prepared according to procedures described previously in the literature.^17*a*,*c*^

### General procedure for the annulation of aziridines 1 and allenoate 2

An oven-dried 10 mL flask was charged with diphenyl-ethylphosphine or diphenyl-*n*-propylphosphine (0.125 mmol), the *N*-4-nitrobenzenesulfonyl-protected aziridine (0.125 mmol), and CH_2_Cl_2_ (5 mL) at room temperature. After adding diethyl 2-vinylidenesuccinate (0.15 mmol) to this solution, the mixture was stirred at room temperature for 48 h. The reaction mixture was concentrated and the residue purified through flash column chromatography (EtOAc/hexane, 1 : 5) to afford the corresponding tetrahydroazepine product.

#### Diethyl *trans*-1-(4-nitrophenylsulfonyl)-3-phenyl-2,3,4,7-tetrahydro-1*H*-azepine-4,5-dicarboxylate (*trans*-4a)

Prepared according to the general procedure as described above catalyzed by EtPPh_2_ in 69% yield (43.3 mg). It was purified by flash chromatography (20% EtOAc/PE) to afford pale-yellow solid. Mp = 132–133 °C; ^1^H NMR (300 MHz, CDCl_3_) *δ* 8.40–8.32 (m, 2H), 7.99–7.92 (m, 2H), 7.31–7.22 (m, 3H), 7.16 (dd, *J* = 7.5, 1.9 Hz, 2H), 7.08 (dd, *J* = 5.0, 2.7 Hz, 1H), 4.58–4.50 (m, 1H), 4.41–4.08 (m, 6H), 3.89–3.78 (m, 1H), 3.59 (dd, *J* = 5.0, 17.9 Hz, 1H), 2.90 (dd, *J* = 11.0, 14.3 Hz, 1H), 1.33 (t, *J* = 7.1 Hz, 3H), 1.18 (t, *J* = 7.1 Hz, 3H); ^13^C NMR (75 MHz, CDCl_3_) *δ* 171.5, 166.2, 150.3, 143.2, 140.4, 136.7, 130.3, 128.8, 128.5, 127.6, 127.4, 124.5, 61.9, 61.5, 51.3, 50.5, 46.9, 46.6, 14.12, 14.06; IR (film) *ν*_max_ 3106, 2983, 2934, 2872, 1715, 1654, 1606, 1532, 1497, 1455, 1401, 1352, 1311, 1245.75, 1166, 1095, 1074, 1048, 1030, 978, 945, 908, 855, 766, 744, 702, 687, 617, 604, 590, 502, 463 cm^−1^; HRMS (ESI) calcd for C_24_H_27_N_2_O_8_S^+^ [M + H]^+^ 503.1483, found 503.1480.

#### Diethyl *trans*-1-(4-nitrophenylsulfonyl)-3-*o*-tolyl-2,3,4,7-tetrahydro-1*H*-azepine-4,5-dicarboxylate (*trans*-4b)

Prepared according to the general procedure as described above catalyzed by *n*-PrPPh_2_ in 36% yield (23.2 mg). It was purified by flash chromatography (20% EtOAc/PE) to afford pale-yellow solid. Mp = 148–149 °C; ^1^H NMR (300 MHz, CDCl_3_) *δ* 8.44–8.28 (m, 2H), 8.04–7.84 (m, 2H), 7.23–7.16 (m, 1H), 7.16–7.08 (m, 2H), 7.07–7.04 (m, 1H), 6.91–6.88 (m, 1H), 4.70–4.64 (m, 1H), 4.62–4.54 (m, 1H), 4.34–4.21 (m, 2H), 4.21–4.05 (m, 3H), 3.75–3.68 (m, 1H), 3.64–3.57 (m, 1H), 2.93–2.85 (m, 1H), 2.50 (s, 3H), 1.34 (t, *J* = 7.1 Hz, 3H), 1.17 (t, *J* = 7.1 Hz, 3H); ^13^C NMR (75 MHz, CDCl_3_) *δ* 171.6, 166.1, 150.2, 143.3, 138.7, 136.8, 136.0, 130.9, 130.1, 128.4, 127.3, 126.3, 125.6, 124.5, 61.8, 61.5, 50.4, 50.3, 46.3, 42.2, 19.7, 14.1, 14.0; IR (film) *ν*_max_ 3105, 2923, 2851, 1716, 1652, 1606, 1531, 1447, 1401, 1351, 1310, 1247, 1166, 1092, 1073, 1047, 1029, 978, 947, 913, 855, 757, 742, 686 cm^−1^; HRMS (ESI) calcd for C_25_H_29_N_2_O_8_S^+^ [M + H]^+^ 517.1639, found 517.1634.

#### Diethyl *trans*-1-(4-nitrophenylsulfonyl)-3-*m*-tolyl-2,3,4,7-tetrahydro-1*H*-azepine-4,5-dicarboxylate (*trans*-4c)

Prepared according to the general procedure as described above catalyzed by *n*-PrPPh_2_ in 33% yield (21.3 mg). It was purified by flash chromatography (20% EtOAc/PE) to afford pale-yellow solid. Mp = 125–126 °C; ^1^H NMR (300 MHz, CDCl_3_) *δ* 8.40–8.30 (m, 2H), 8.07–7.92 (m, 2H), 7.17–7.12 (m, 1H), 7.10–7.01 (m, 2H), 7.00–6.90 (m, 2H), 4.58–4.51 (m, 1H), 4.36–4.01 (m, 6H), 3.85–3.78 (m, 1H), 3.58 (dd, *J* = 5.0, 17.9 Hz, 1H), 2.88 (dd, *J* = 11.1, 14.3 Hz, 1H), 2.28 (s, 3H), 1.33 (t, *J* = 7.1 Hz, 3H), 1.19 (t, *J* = 7.1 Hz, 3H); ^13^C NMR (75 MHz, CDCl_3_) *δ* 171.5, 166.2, 150.2, 143.2, 140.4, 138.4, 136.6, 130.4, 128.7, 128.5, 128.34, 128.30, 128.2, 124.5, 124.2, 61.9, 61.5, 51.3, 50.5, 46.9, 46.6, 21.3, 14.11, 14.09; IR (film) *ν*_max_ 3106, 2982, 2932, 1715, 1653, 1607, 1532, 1447, 1401, 1351, 1311, 1253, 1166, 1093, 1074, 1049, 1029, 978, 947, 913, 856, 821, 795, 765, 742, 703, 686, 607, 463 cm^−1^; HRMS (ESI) calcd for C_25_H_29_N_2_O_8_S^+^ [M + H]^+^ 517.1639, found 517.1631.

#### Diethyl *trans*-1-(4-nitrophenylsulfonyl)-3-*p*-tolyl-2,3,4,7-tetrahydro-1*H*-azepine-4,5-dicarboxylate (*trans*-4d)

Prepared according to the general procedure as described above catalyzed by *n*-PrPPh_2_ in 54% yield (34.9 mg). It was purified by flash chromatography (20% EtOAc/PE) to afford pale-yellow solid. Mp = 118–119 °C; ^1^H NMR (300 MHz, CDCl_3_) *δ* 8.38–8.33 (m, 2H), 8.05–7.87 (m, 2H), 7.19–6.91 (m, 5H), 4.60–4.46 (m, 1H), 4.39–4.06 (m, 6H), 3.84–3.77 (m, 1H), 3.62–3.54 (m, 1H), 2.92–2.83 (m, 11.0 Hz, 1H), 2.29 (s, 3H), 1.33 (t, *J* = 7.1 Hz, 3H), 1.20 (t, *J* = 7.1 Hz, 3H); ^13^C NMR (75 MHz, CDCl_3_) *δ* 171.5, 166.2, 150.2, 143.2, 137.4, 137.3, 136.6, 130.4, 129.4, 128.5, 127.2, 124.5, 61.8, 61.5, 51.4, 50.5, 46.7, 46.5, 21.0, 14.11, 14.06; IR (film) *ν*_max_ 3105, 3057, 2984, 2960, 2927, 2853, 2307, 1715, 1655, 1607, 1533, 1516, 1464, 1447, 1402, 1351, 1310, 1266, 1167, 1093, 1074, 1049, 1029, 978, 946, 911, 880, 856, 819, 801, 742, 704, 687, 609, 590, 556, 522, 463 cm^−1^; HRMS (ESI) calcd for C_25_H_29_N_2_O_8_S^+^ [M + H]^+^ 517.1639, found 517.1630.

#### Diethyl *trans*-3-(2,4-dimethylphenyl)-1-(4-nitrophenylsulfonyl)-2,3,4,7-tetrahydro-1*H*-azepine-4,5-dicarboxylate (*trans*-4e)

Prepared according to the general procedure as described above catalyzed by EtPPh_2_ in 54% yield (35.8 mg). It was purified by flash chromatography (20% EtOAc/PE) to afford pale-yellow solid. Mp = 121–122 °C; ^1^H NMR (300 MHz, CDCl_3_) *δ* 8.39–8.33 (m, 2H), 7.98–7.92 (m, 2H), 7.11–7.08 (m, 1H), 7.01–7.00 (m, 1H), 6.87–6.84 (m, 1H), 6.79–6.76 (m, 1H), 4.67–4.50 (m, 2H), 4.31–4.21 (m, 2H), 4.20–4.09 (m, 2H), 4.07–4.06 (m, 1H), 3.73–3.55 (m, 2H), 2.87 (dd, *J* = 11.4, 14.2 Hz, 1H), 2.46 (s, 3H), 2.25 (s, 3H), 1.34 (t, *J* = 7.1 Hz, 3H), 1.19 (t, *J* = 7.1 Hz, 3H); ^13^C NMR (75 MHz, CDCl_3_) *δ* 171.7, 166.2, 150.2, 143.3, 136.9, 136.8, 135.8, 135.7, 131.6, 130.1, 128.4, 126.9, 125.5, 124.5, 61.8, 61.5, 50.44, 50.36, 46.5, 41.9, 20.8, 19.6, 14.1, 14.0; IR (film) *ν*_max_ 2963, 2926, 2854, 1719, 1606, 1532, 1448, 1401, 1351, 1310, 1260, 1167, 1092, 1028, 978, 913, 855, 801, 754, 744, 686, 610, 463 cm^−1^; HRMS (ESI) calcd for C_26_H_31_N_2_O_8_S^+^ [M + H]^+^ 531.1796, found 531.1789.

#### Diethyl *trans*-3-(2,5-dimethylphenyl)-1-(4-nitrophenylsulfonyl)-2,3,4,7-tetrahydro-1*H*-azepine-4,5-dicarboxylate (*trans*-4f)

Prepared according to the general procedure as described above catalyzed by *n*-PrPPh_2_ in 35% yield (23.2 mg). It was purified by flash chromatography (20% EtOAc/PE) to afford pale-yellow solid. Mp = 130–131 °C; ^1^H NMR (300 MHz, CDCl_3_) *δ* 8.40–8.32 (m, 2H), 7.99–7.92 (m, 2H), 7.14–7.03 (m, 2H), 6.94–6.91 (m, 1H), 6.69–6.68 (m, 1H), 4.68–4.52 (m, 2H), 4.31–4.21 (m, 2H), 4.19–4.12 (m, 2H), 4.07–4.06 (m, 1H), 3.75–3.55 (m, 2H), 2.89 (dd, *J* = 11.5, 14.2 Hz, 1H), 2.45 (s, 3H), 2.17 (s, 3H), 1.34 (t, *J* = 7.1 Hz, 3H), 1.19 (t, *J* = 7.1 Hz, 3H); ^13^C NMR (75 MHz, CDCl_3_) *δ* 171.6, 166.2, 150.1, 143.2, 138.5, 136.8, 135.5, 132.7, 130.7, 130.1, 128.4, 127.9, 126.3, 124.4, 61.7, 61.4, 50.3, 46.3, 42.1, 20.9, 19.1, 14.02, 14.01; IR (film) *ν*_max_ 2981, 2928, 1714, 1651, 1606, 1531, 1504, 1447, 1401, 1351, 1311, 1249, 1165, 1092, 1073, 1047, 977, 947, 913, 856, 831, 754, 739, 714, 686, 607 cm^−1^; HRMS (ESI) calcd for C_26_H_31_N_2_O_8_S^+^ [M + H]^+^ 531.1796, found 531.1790.

#### Diethyl *trans*-3-mesityl-1-(4-nitrophenylsulfonyl)-2,3,4,7-tetrahy-dro-1*H*-azepine-4,5-dicarboxylate (*trans*-4g)

Prepared according to the general procedure as described above catalyzed by *n*-PrPPh_2_ in 39% yield (26.6 mg). It was purified by flash chromatography (20% EtOAc/PE) to afford pale-yellow solid. Mp = 130–131 °C; ^1^H NMR (300 MHz, CDCl_3_) *δ* 8.44–8.24 (m, 2H), 8.03–7.86 (m, 2H), 7.05–7.02 (m, 1H), 6.84–6.81 (m, 2H), 4.47–4.39 (m, 1H), 4.33–4.05 (m, 5H), 3.97 (q, *J* = 7.1 Hz, 2H), 3.56–3.44 (m, 1H), 3.40–3.33 (m, 1H), 2.38 (s, 3H), 2.25 (s, 3H), 2.23 (s, 3H), 1.25 (t, *J* = 7.1 Hz, 3H), 1.10 (t, *J* = 7.1 Hz, 3H); ^13^C NMR (75 MHz, CDCl_3_) *δ* 172.5, 166.7, 150.2, 144.2, 136.9, 135.8, 134.0, 133.3, 131.0, 129.4, 128.3, 124.5, 61.5, 61.3, 49.2, 47.1, 46.9, 42.1, 21.2, 21.1, 20.6, 14.1, 13.8; IR (film) *ν*_max_ 3105, 2982, 2936, 2872, 1730, 1655, 1608, 1532, 1448, 1401, 1350, 1310, 1246, 1165, 1096, 1030, 957, 928, 855, 754, 740, 686, 612, 579, 463 cm^−1^; HRMS (ESI) calcd for C_27_H_33_N_2_O_8_S^+^ [M + H]^+^ 545.1952, found 545.1929.

#### Diethyl *trans*-3-(4-*tert*-butylphenyl)-1-(4-nitrophenylsulfonyl)-2,3,4,7-tetrahydro-1*H*-azepine-4,5-dicarboxylate (*trans*-4h)

Prepared according to the general procedure as described above catalyzed by *n*-PrPPh_2_ in 37% yield (25.8 mg). It was purified by flash chromatography (20% EtOAc/PE) to afford pale-yellow semi-solid. ^1^H NMR (300 MHz, CDCl_3_) *δ* 8.42–8.26 (m, 2H), 8.00–7.86 (m, 2H), 7.37–7.19 (m, 2H), 7.10–7.07 (m, 3H), 4.57–4.51 (m, 1H), 4.38–4.06 (m, 6H), 3.85–3.78 (m, 1H), 3.62–3.54 (m, 1H), 2.92–2.84 (m, 1H), 1.32 (t, *J* = 7.1 Hz, 3H), 1.27 (s, 9H), 1.18 (t, *J* = 7.1 Hz, 3H); ^13^C NMR (75 MHz, CDCl_3_) *δ* 171.4, 166.2, 150.2, 143.2, 137.7, 136.8, 133.3, 132.7, 130.3, 128.6, 128.4, 127.7, 127.6, 126.4, 126.2, 126.0, 125.3, 124.5, 61.9, 61.6, 51.1, 50.5, 46.9, 46.6, 14.1, 14.0; IR (film) *ν*_max_ 3105, 3061, 2982, 2936, 2872, 1715, 1654, 1604, 1531, 1446, 1401, 1351, 1310, 1249, 1166, 1093, 1074, 1048, 1029, 977, 946, 915, 900, 856, 822, 741, 686, 624, 607, 589, 479, 463 cm^−1^; HRMS (ESI) calcd for C_28_H_35_N_2_O_8_S^+^ [M + H]^+^ 559.2109, found 559.2106.

#### Diethyl *trans*-3-(2-fluorophenyl)-1-(4-nitrophenylsulfonyl)-2,3,4,7-tetrahydro-1*H*-azepine-4,5-dicarboxylate (*trans*-4i)

Prepared according to the general procedure as described above catalyzed by *n*-PrPPh_2_ in 29% yield (18.9 mg). It was purified by flash chromatography (20% EtOAc/PE) to afford pale-yellow semi-solid. ^1^H NMR (300 MHz, CDCl_3_) *δ* 8.42–8.32 (m, 2H), 8.00–7.92 (m, 2H), 7.25–7.18 (m, 1H), 7.18–6.96 (m, 4H), 4.66–4.58 (m, 1H), 4.53–4.47 (m, 1H), 4.28–4.13 (m, 5H), 3.84–3.77 (m, 1H), 3.70–3.62 (m, 1H), 3.05–2.96 (m, 1H), 1.31 (t, *J* = 7.1 Hz, 3H), 1.21 (t, *J* = 7.1 Hz, 3H); ^13^C NMR (75 MHz, CDCl_3_) *δ* 171.3, 166.2, 160.6 (d, *J* = 246.7 Hz), 150.3, 143.3, 136.6, 130.5, 129.2 (d, *J* = 8.5 Hz), 128.7 (d, *J* = 4.4 Hz), 128.5, 127.2 (d, *J* = 14.4 Hz), 124.5, 124.4 (d, *J* = 3.5 Hz), 115.9 (d, *J* = 22.7 Hz), 61.9, 61.6, 49.93, 49.90, 45.9, 40.6, 14.1; IR (film) *ν*_max_ 3106, 2983, 2931, 1716, 1606, 1586, 1532, 1492, 1455, 1401, 1351, 1310, 1248, 1167, 1094, 1048, 1029, 979, 946, 913, 856, 818, 757, 744, 686 cm^−1^; HRMS (ESI) calcd for C_24_H_26_FN_2_O_8_S^+^ [M + H]^+^ 521.1388, found 521.1389.

#### Diethyl *trans*-3-(3-fluorophenyl)-1-(4-nitrophenylsulfonyl)-2,3,4,7-tetrahydro-1*H*-azepine-4,5-dicarboxylate (*trans*-4j)

Prepared according to the general procedure as described above catalyzed by *n*-PrPPh_2_ in 32% yield (20.8 mg). It was purified by flash chromatography (20% EtOAc/PE) to afford pale-yellow semi-solid. ^1^H NMR (300 MHz, CDCl_3_) *δ* 8.39–8.35 (m, 2H), 8.02–7.91 (m, 2H), 7.30–6.82 (m, 5H), 4.59–4.51 (m, 1H), 4.42–4.09 (m, 6H), 3.88–3.81 (m, 1H), 3.58 (dd, *J* = 18.0, 5.0 Hz, 1H), 2.90–2.82 (m, 1H), 1.34 (t, *J* = 7.1 Hz, 3H), 1.21 (t, *J* = 7.1 Hz, 3H); ^13^C NMR (75 MHz, CDCl_3_) *δ* 171.1, 166.0, 162.8 (d, *J* = 246.8 Hz), 150.3, 143.1, 142.9 (d, *J* = 7.0 Hz), 136.9, 130.3 (d, *J* = 8.3 Hz), 129.9, 128.4, 124.5, 123.1 (d, *J* = 2.8 Hz), 114.5 (d, *J* = 16.0 Hz), 114.2 (d, *J* = 16.8 Hz), 62.0, 61.6, 50.9, 50.6, 46.5, 46.2, 14.1, 14.0; IR (film) *ν*_max_ 2983, 1719, 1590, 1532, 1449, 1351, 1253, 1167, 1095, 857, 742, 596 cm^−1^; HRMS (ESI) calcd for C_24_H_26_FN_2_O_8_S^+^ [M + H]^+^ 521.1388, found 521.1384.

#### Diethyl *trans*-3-(4-fluorophenyl)-1-(4-nitrophenylsulfonyl)-2,3,4,7-tetrahydro-1*H*-azepine-4,5-dicarboxylate (*trans*-4k)

Prepared according to the general procedure as described above catalyzed by *n*-PrPPh_2_ in 37% yield (24.1 mg). It was purified by flash chromatography (20% EtOAc/PE) to afford pale-yellow semi-solid. ^1^H NMR (300 MHz, CDCl_3_) *δ* 8.46–8.26 (m, 2H), 8.08–7.87 (m, 2H), 7.39–7.06 (m, 3H), 7.06–6.89 (m, 2H), 4.61–4.48 (m, 1H), 4.43–4.09 (m, 6H), 3.92–3.74 (m, 1H), 3.63–3.56 (m, 1H), 2.90–2.82 (m, 1H), 1.33 (t, *J* = 7.1 Hz, 3H), 1.20 (t, *J* = 7.1 Hz, 3H); ^13^C NMR (75 MHz, CDCl_3_) *δ* 171.3, 166.1, 162.1 (d, *J* = 246.5 Hz), 150.3, 143.1, 136.9, 136.2 (d, *J* = 3.3 Hz), 130.0, 129.0 (d, *J* = 8.0 Hz), 128.4, 124.54, 124.51, 115.6 (d, *J* = 21.3 Hz), 61.9, 61.6, 51.2, 50.5, 46.6, 46.1, 14.1, 14.0; IR (film) *ν*_max_ 2983, 1717, 1606, 1532, 1511, 1352, 1244, 1166, 1092, 1048, 856, 743, 608 cm^−1^; HRMS (ESI) calcd for C_24_H_26_FN_2_O_8_S^+^ [M + H]^+^ 521.1388, found 521.1388.

#### Diethyl *trans*-3-(2-chlorophenyl)-1-(4-nitrophenylsulfonyl)-2,3,4,7-tetrahydro-1*H*-azepine-4,5-dicarboxylate (*trans*-4l)

Prepared according to the general procedure as described above catalyzed by *n*-PrPPh_2_ in 48% yield (32.2 mg). It was purified by flash chromatography (20% EtOAc/PE) to afford pale-yellow semi-solid. ^1^H NMR (300 MHz, CDCl_3_) *δ* 8.46–8.26 (m, 2H), 8.08–7.87 (m, 2H), 7.39–7.06 (m, 3H), 7.06–6.89 (m, 2H), 4.61–4.48 (m, 1H), 4.43–4.09 (m, 6H), 3.92–3.74 (m, 1H), 3.63–3.56 (m, 1H), 2.90–2.82 (m, 1H), 1.33 (t, *J* = 7.1 Hz, 3H), 1.20 (t, *J* = 7.1 Hz, 3H); ^13^C NMR (75 MHz, CDCl_3_) *δ* 171.2, 166.1, 150.3, 143.3, 138.0, 137.1, 133.9, 130.8, 130.2, 130.1, 128.8, 128.6, 128.5, 127.6, 127.1, 124.5, 61.9, 61.6, 50.2, 49.8, 45.7, 42.5, 14.0, 13.7; IR (film) *ν*_max_ 2983, 1717, 1606, 1532, 1511, 1352, 1244, 1166, 1092, 1048, 856, 743, 608 cm^−1^; HRMS (ESI) calcd for C_24_H_26_ClN_2_O_8_S^+^ [M + H]^+^ 537.1093, found 537.1093.

#### Diethyl *trans*-3-(2-bromophenyl)-1-(4-nitrophenylsulfonyl)-2,3,4,7-tetrahydro-1*H*-azepine-4,5-dicarboxylate (*trans*-4m)

Prepared according to the general procedure as described above catalyzed by *n*-PrPPh_2_ in 18% yield (13.1 mg). It was purified by flash chromatography (20% EtOAc/PE) to afford pale-yellow semi-solid. ^1^H NMR (300 MHz, CDCl_3_) *δ* 8.48–8.22 (m, 2H), 8.10–7.86 (m, 2H), 7.60–7.57 (m, 1H), 7.22–6.93 (m, 4H), 4.97–4.91 (m, 1H), 4.57–4.51 (m, 1H), 4.38–4.02 (m, 5H), 3.90–3.83 (m, 1H), 3.68–3.61 (m, 1H), 2.85–2.77 (m, 1H), 1.34 (t, *J* = 7.1 Hz, 3H), 1.21 (t, *J* = 7.1 Hz, 3H); ^13^C NMR (75 MHz, CDCl_3_) *δ* 171.1, 166.1, 150.2, 143.2, 139.6, 137.3, 133.4, 129.9, 128.9, 128.5, 127.7, 127.6, 124.6, 124.5, 61.9, 61.6, 50.3, 49.9, 45.7, 45.2, 14.0; IR (film) *ν*_max_ 3105, 2962, 2928, 2872, 1720, 1654, 1606, 1531, 1471, 1445, 1401, 1351, 1310, 1257, 1167, 1093, 1075, 1049, 1024, 979, 947, 913, 855, 763, 745, 734, 686, 666 cm^−1^; HRMS (ESI) calcd for C_24_H_26_BrN_2_O_8_S^+^ [M + H]^+^ 581.0588, found 581.0593.

#### Diethyl *trans*-3-(naphthalen-2-yl)-1-(4-nitrophenylsulfonyl)-2,3,4,7-tetrahydro-1*H*-azepine-4,5-dicarboxylate (*trans*-4n)

Prepared according to the general procedure as described above catalyzed by *n*-PrPPh_2_ in 36% yield (24.9 mg). It was purified by flash chromatography (20% EtOAc/PE) to afford pale-yellow semi-solid. ^1^H NMR (300 MHz, CDCl_3_) *δ* 8.43–8.23 (m, 2H), 8.03–7.88 (m, 2H), 7.84–7.67 (m, 3H), 7.62 (s, 1H), 7.50–7.39 (m, 2H), 7.31–7.22 (m, 1H), 7.14–7.12 (m, 1H), 4.60–4.50 (m, 2H), 4.32–4.25 (m, 3H), 4.14 (q, *J* = 7.1 Hz, 2H), 3.94–3.87 (m, 1H), 3.68–3.61 (m, 1H), 3.09–3.00 (m, 1H), 1.34 (t, *J* = 7.1 Hz, 3H), 1.14 (t, *J* = 7.1 Hz, 3H); ^13^C NMR (75 MHz, CDCl_3_) *δ* 171.4, 166.2, 150.2, 143.2, 137.7, 136.8, 133.3, 132.7, 130.3, 128.6, 128.4, 127.7, 127.6, 126.4, 126.2, 126.0, 125.3, 124.5, 61.9, 61.6, 51.1, 50.5, 46.9, 46.6, 14.1, 14.0; IR (film) *ν*_max_ 3105, 3061, 2982, 2936, 2872, 1715, 1654, 1604, 1531, 1446, 1401, 1351, 1310, 1249, 1166, 1093, 1074, 1048, 1029, 977, 946, 915, 900, 856, 822, 741, 686, 624, 607, 589, 479, 463 cm^−1^; HRMS (ESI) calcd for C_28_H_29_N_2_O_8_S^+^ [M + H]^+^ 553.1639, found 553.1631.

## Conflicts of interest

There are no conflicts to declare.

## Supplementary Material

RA-009-C8RA09852B-s001

RA-009-C8RA09852B-s002
