# Supplementary material for: Phosphine-promoted [4 + 3] annulation of allenoate with aziridines for synthesis of tetrahydroazepines: phosphine-dependent [3 + 3] and [4 + 3] pathways
Source: RSC Adv. 2019 Jan 9;9(3):1214–21. doi: 10.1039/c8ra09852b (PMC6764531; doi:10.1039/c8ra09852b)
Supplement: RA-009-C8RA09852B-s001 [file RA-009-C8RA09852B-s001.pdf]

Electronic Supplementary Material (ESI) for RSC Advances.
This journal is © The Royal Society of Chemistry 2018

Electronic Supplementary Information

Phosphine-Promoted [4 + 3] Annulation of Allenoate with Aziridines for Synthesis of Tetrahydroazepines: Phosphine-Dependent [3 + 3] and [4 + 3] Pathways

Honglei Liu,[†] Yan Lin,[†] Yan Zhao,[†] Miaoren Xiao,[‡] Leijie Zhou,[†] Qijun Wang,[†] Cheng Zhang,[†] Dongqi Wang,[‡] Ohyun Kwon*[,ξ] and Hongchao Guo*[,†]

[†]Department of Applied Chemistry, China Agricultural University, 2 West Yuanmingyuan Road, Beijing 100193, P. R. China

[‡]Institute of High Energy Physics, Chinese Academy of Science, 19B Yuquan Lu, Shijingshan District, Beijing 100049, P. R. China

[ξ]Department of Chemistry and Biochemistry, University of California, Los Angeles, California 90095-1569

Email: hchguo@cau.edu.cn, ohyun@chem.ucla.edu

Contents

^{1}H NMR Spectra of Substrates 1 and 2 S3

^{1}H and ^{13}C NMR Spectra of All Products 4 S12

X-Ray Crystallographic Information S26

^{1}H NMR Spectra of Substrates 1 and 2

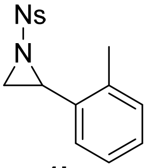

1b

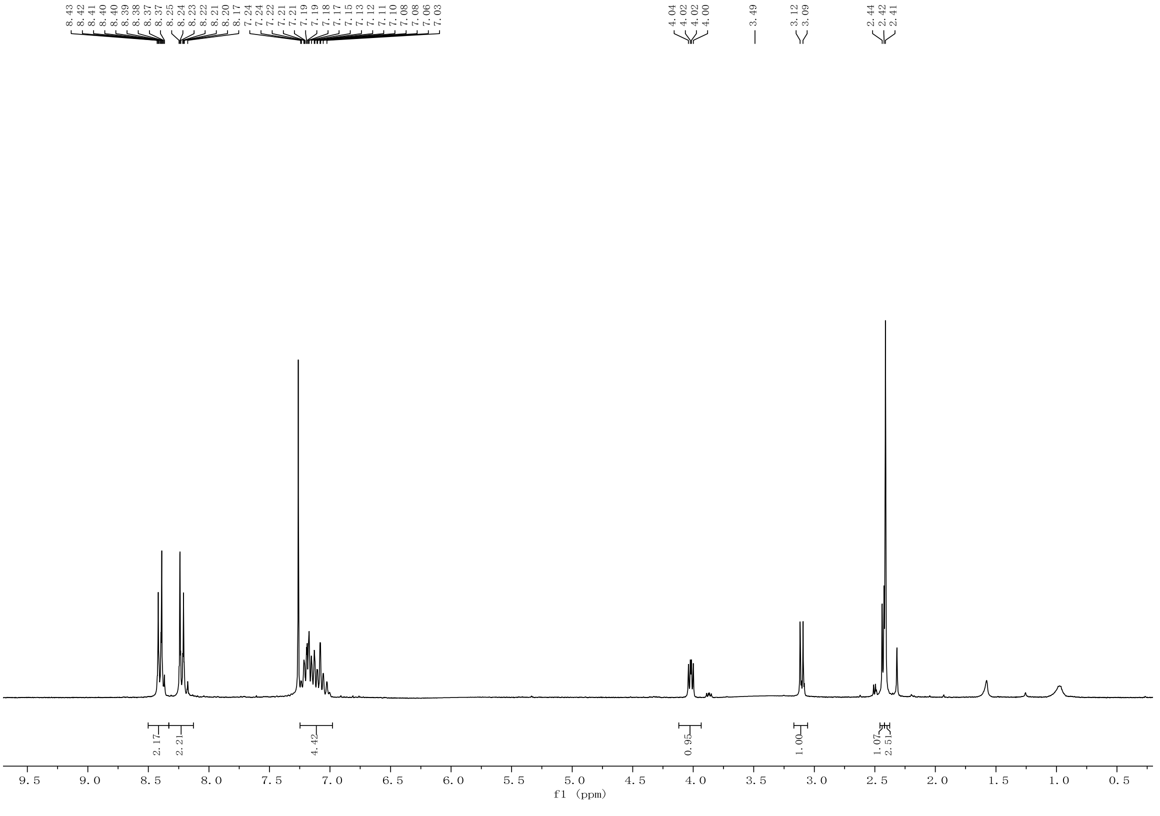

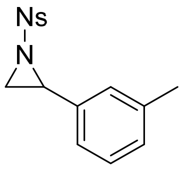
Ns
N

1c

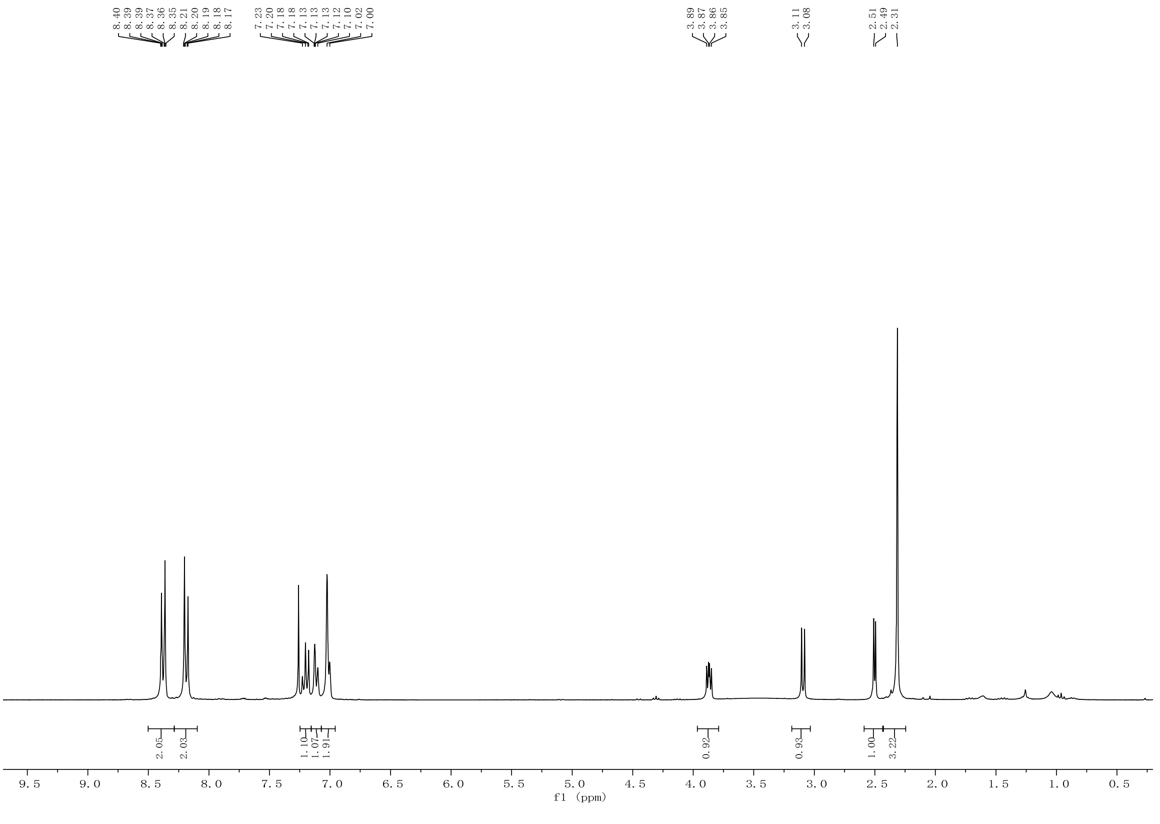
8.40
8.39
8.39
8.37
8.36
8.35
8.21
8.20
8.19
8.18
8.17
7.23
7.20
7.18
7.18
7.13
7.13
7.13
7.13
7.12
7.10
7.02
7.00
3.89
3.87
3.86
3.85
3.11
3.08
2.51
2.49
2.31
2.05
2.03
1.10
1.07
1.91
0.92
0.93
1.00
3.22
9.5
9.0
8.5
8.0
7.5
7.0
6.5
6.0
5.5
5.0
4.5
4.0
3.5
3.0
2.5
2.0
1.5
1.0
0.5
f1 (ppm)

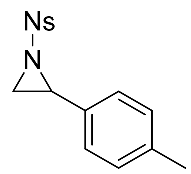
Ns
N

1d

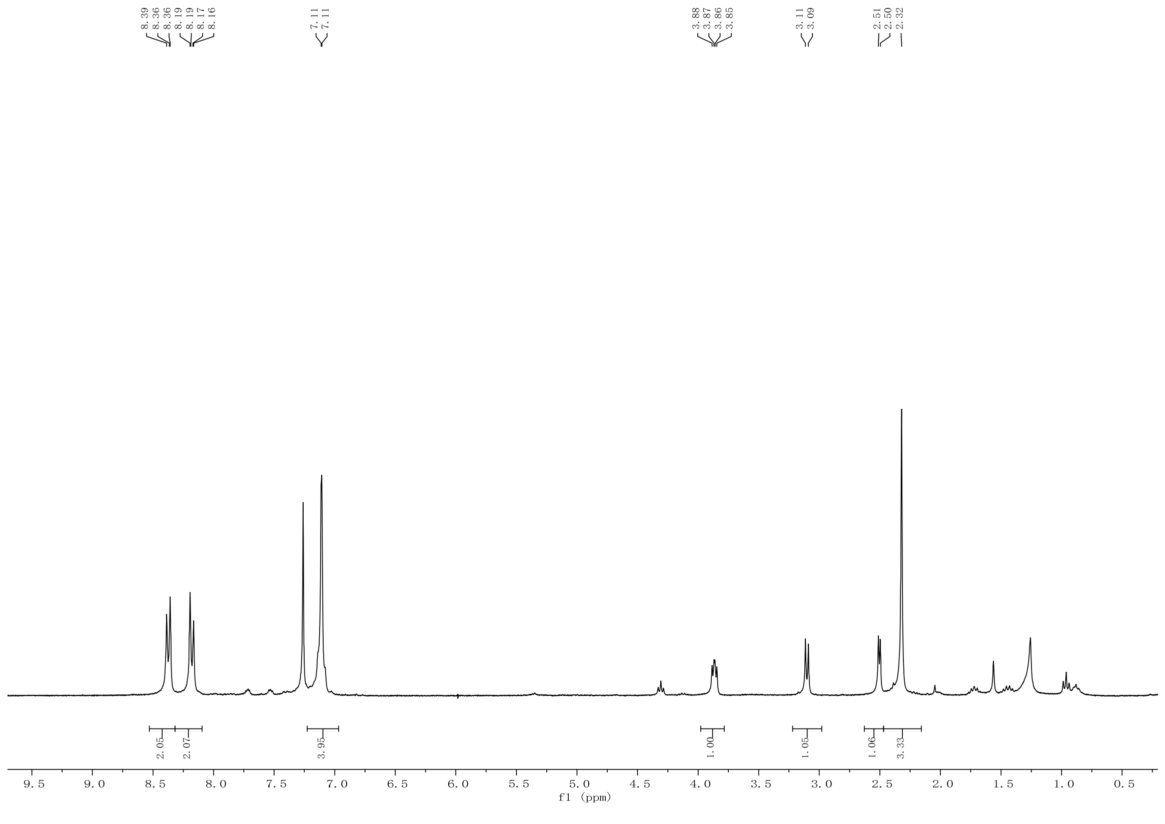
8.39
8.36
8.36
8.19
8.19
8.17
8.16
7.11
7.11
3.88
3.87
3.86
3.85
3.11
3.09
2.51
2.50
2.32
2.05
2.07
3.95
1.00
1.05
1.06
3.33
9.5 9.0 8.5 8.0 7.5 7.0 6.5 6.0 5.5 5.0 4.5 4.0 3.5 3.0 2.5 2.0 1.5 1.0 0.5
f1 (ppm)

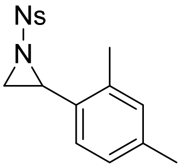
Ns
N

1e

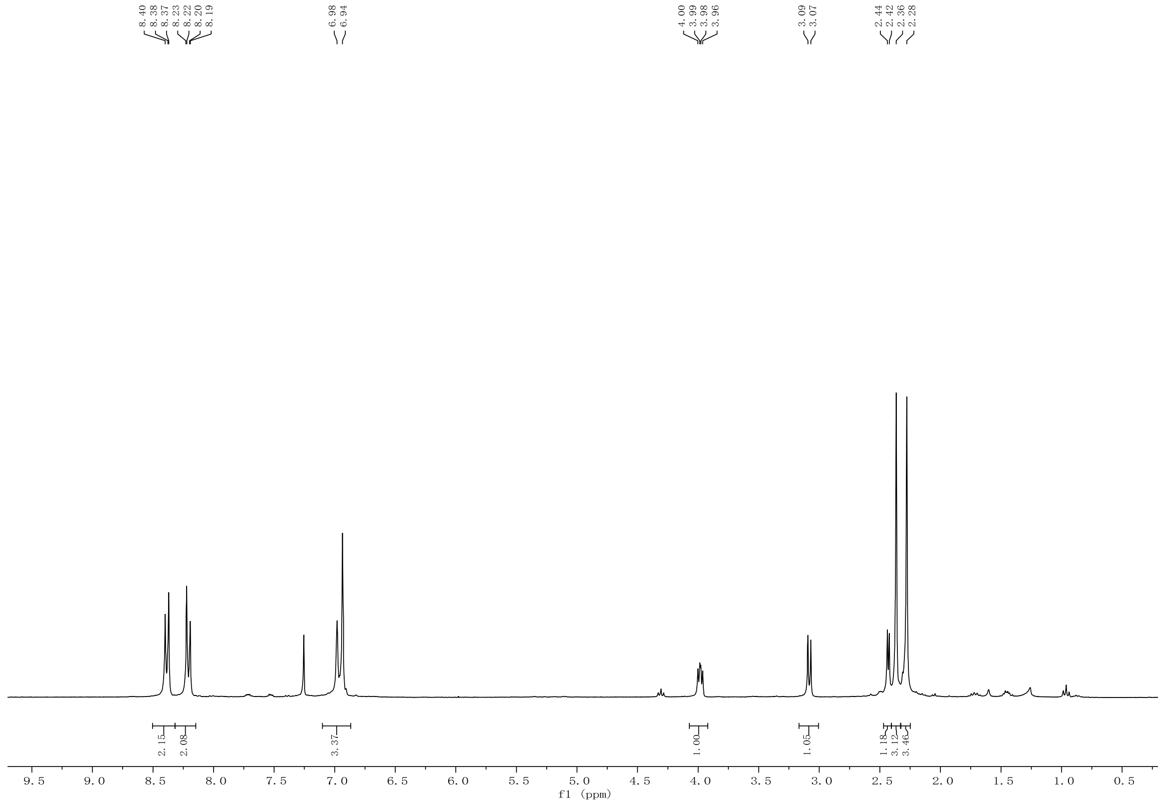
8.40
8.38
8.37
8.23
8.22
8.20
8.19
6.98
6.94
4.00
3.99
3.98
3.96
3.09
3.07
2.44
2.42
2.36
2.28
2.15
2.08
3.37
1.00
1.05
1.18
3.12
3.46
9.5
9.0
8.5
8.0
7.5
7.0
6.5
6.0
5.5
5.0
4.5
4.0
3.5
3.0
2.5
2.0
1.5
1.0
0.5
f1 (ppm)

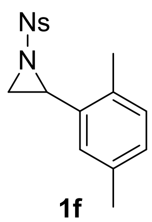

1f

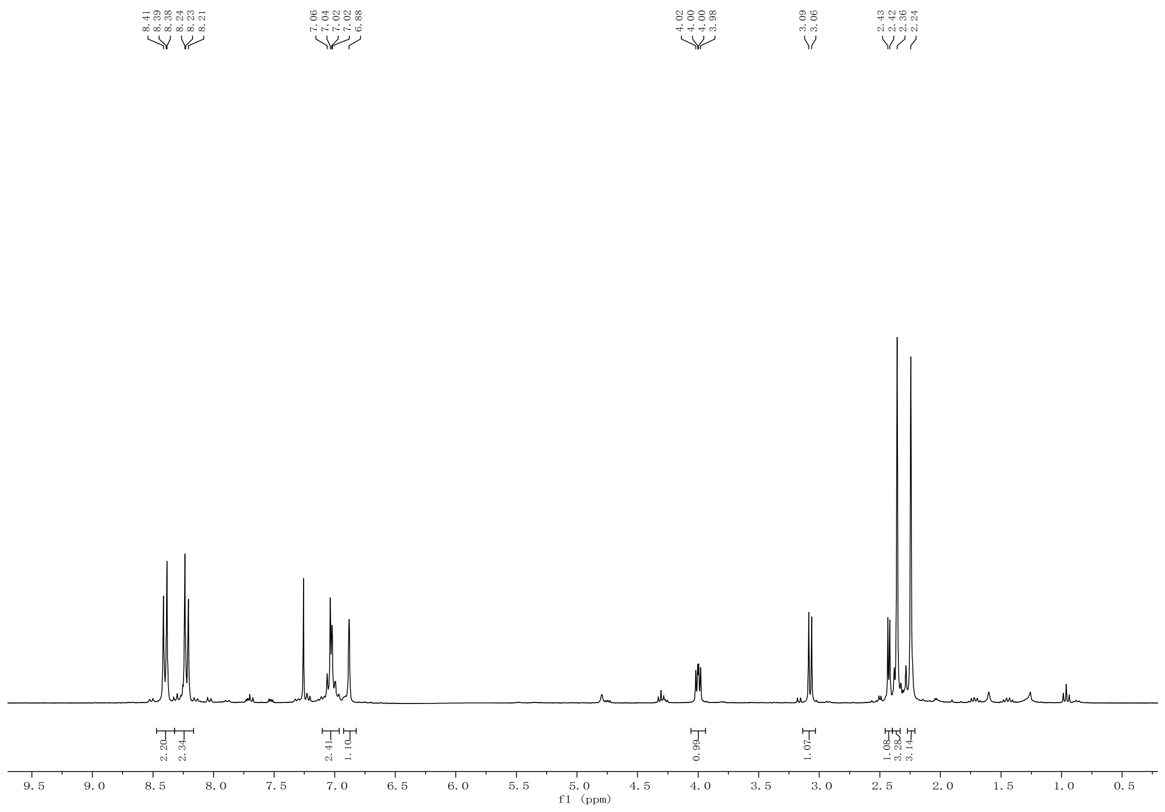
8.41
8.39
8.38
8.24
8.23
8.21
7.06
7.04
7.02
7.02
6.88
4.02
4.00
4.00
3.98
3.09
3.06
2.43
2.42
2.36
2.24
2.20
2.34
2.41
1.10
0.99
1.07
1.08
3.28
3.14
9.5
9.0
8.5
8.0
7.5
7.0
6.5
6.0
5.5
5.0
4.5
4.0
3.5
3.0
2.5
2.0
1.5
1.0
0.5
f1 (ppm)

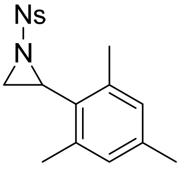

1g

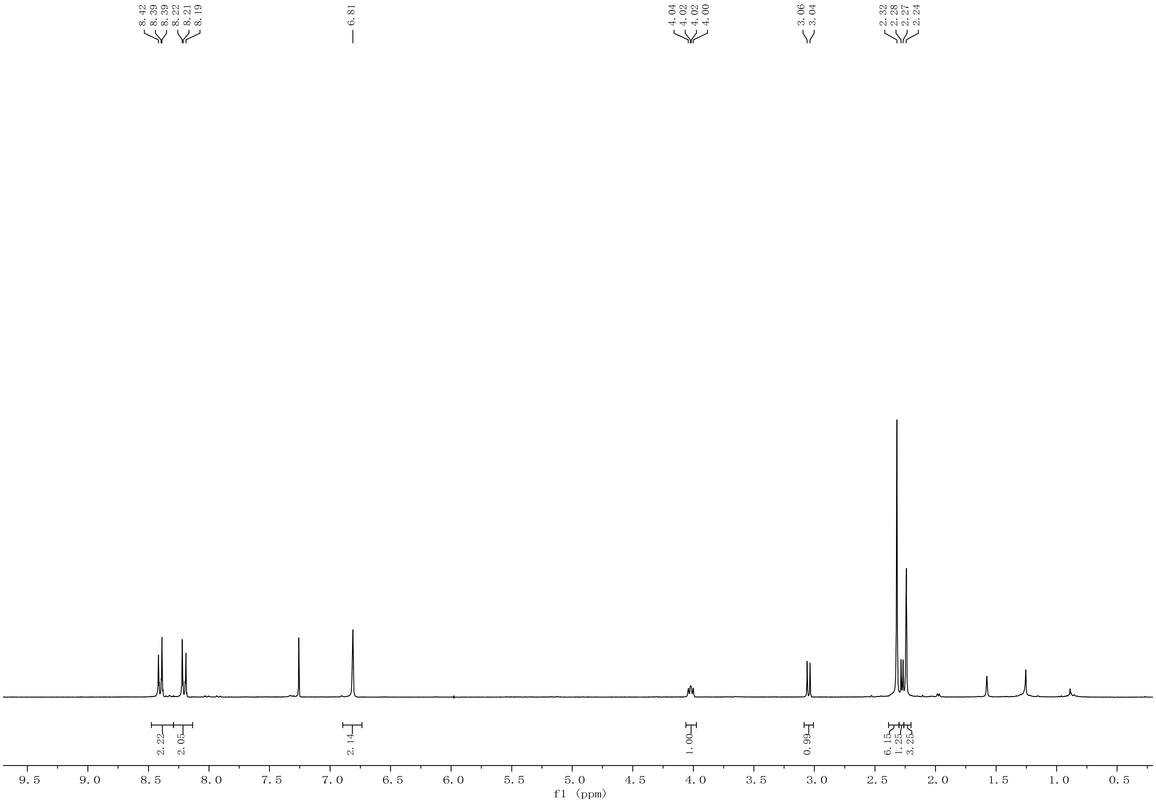
8.42
8.39
8.39
8.22
8.21
8.19
6.81
4.04
4.02
4.02
4.00
3.06
3.04
2.32
2.28
2.27
2.24
2.22
2.05
2.14
1.00
0.99
6.15
1.25
3.25
9.5
9.0
8.5
8.0
7.5
7.0
6.5
6.0
5.5
5.0
4.5
4.0
3.5
3.0
2.5
2.0
1.5
1.0
0.5
f1 (ppm)

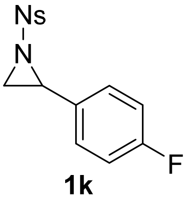
Ns
N
F

1k

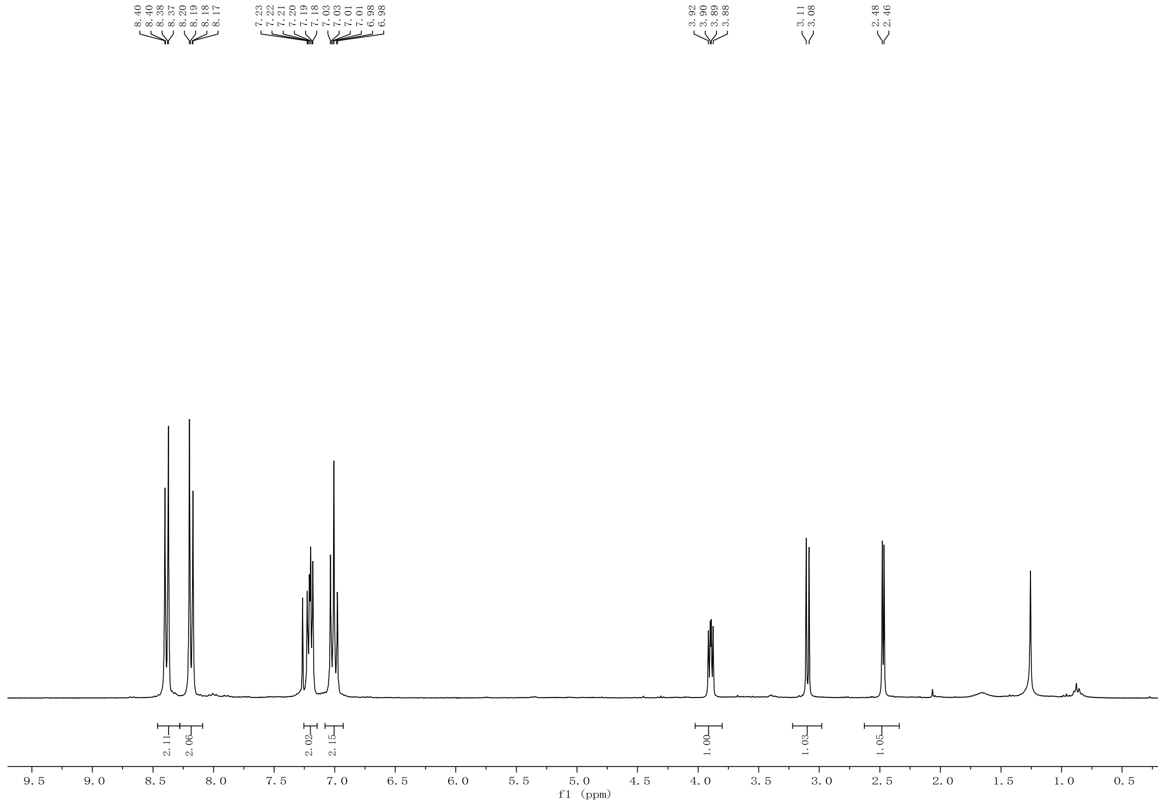
8.40
8.40
8.38
8.37
8.20
8.19
8.18
8.17
7.23
7.22
7.21
7.20
7.19
7.18
7.03
7.03
7.01
7.01
6.98
6.98
3.92
3.90
3.89
3.88
3.11
3.08
2.48
2.46
2.11
2.06
2.02
2.15
1.00
1.03
1.05
9.5
9.0
8.5
8.0
7.5
7.0
6.5
6.0
5.5
5.0
4.5
4.0
3.5
3.0
2.5
2.0
1.5
1.0
0.5
f1 (ppm)

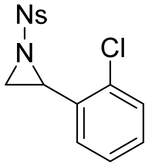

1l

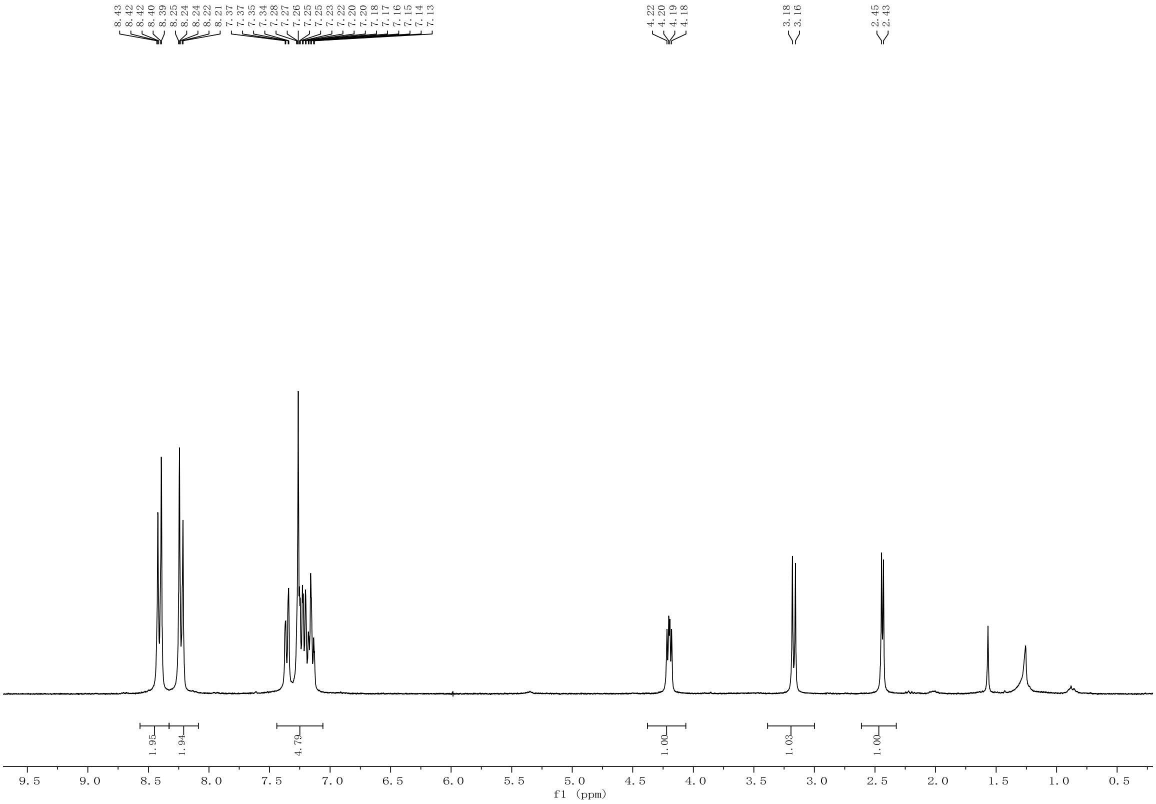

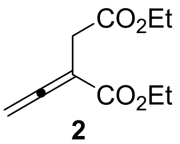
CO2Et
CO2Et

2

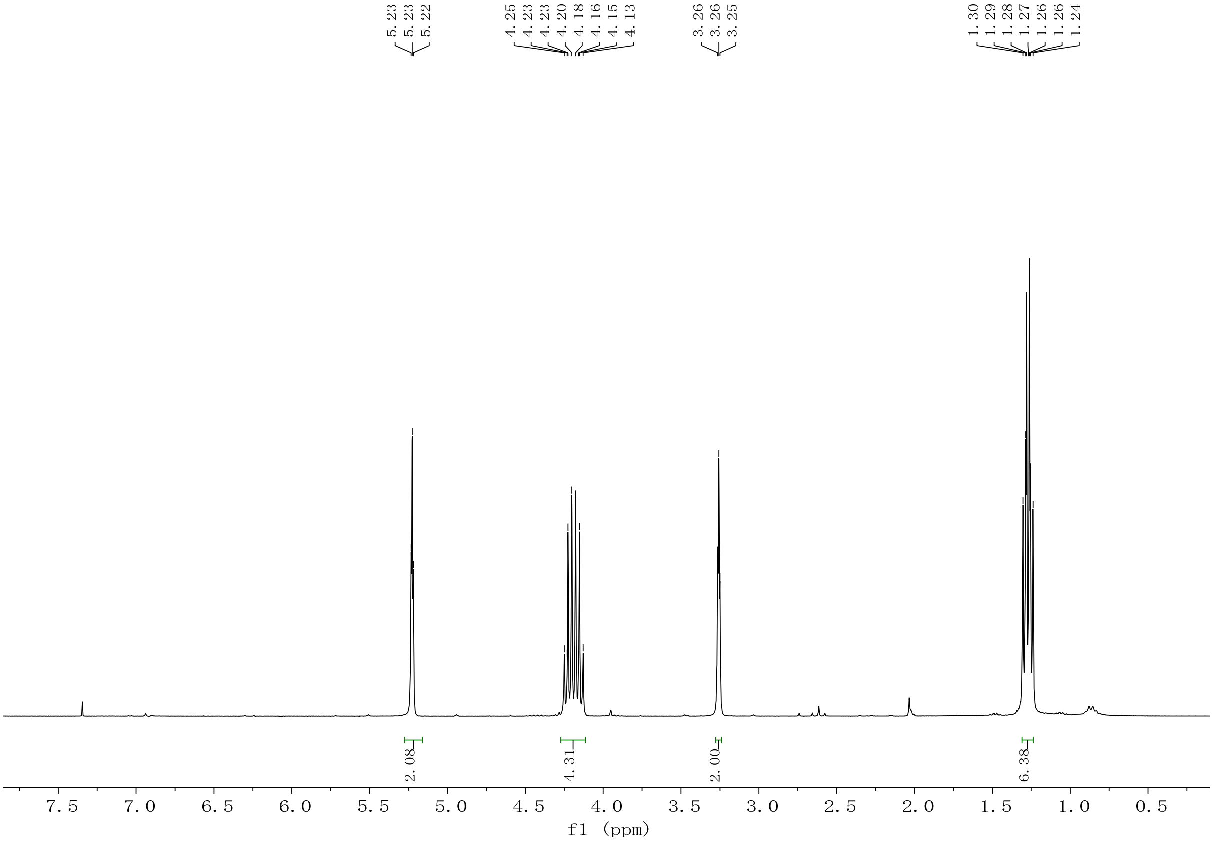
5.23
5.23
5.22
4.25
4.23
4.23
4.20
4.18
4.16
4.15
4.13
3.26
3.26
3.25
1.30
1.29
1.28
1.27
1.26
1.26
1.24
2.08
4.31
2.00
6.38
7.5 7.0 6.5 6.0 5.5 5.0 4.5 4.0 3.5 3.0 2.5 2.0 1.5 1.0 0.5
f1 (ppm)

^{1}H and ^{13}C NMR Spectra of All Products 4

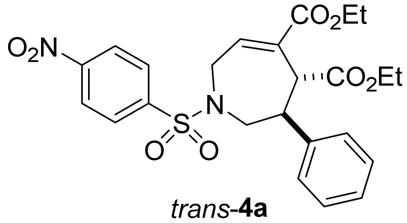

trans-**4a**

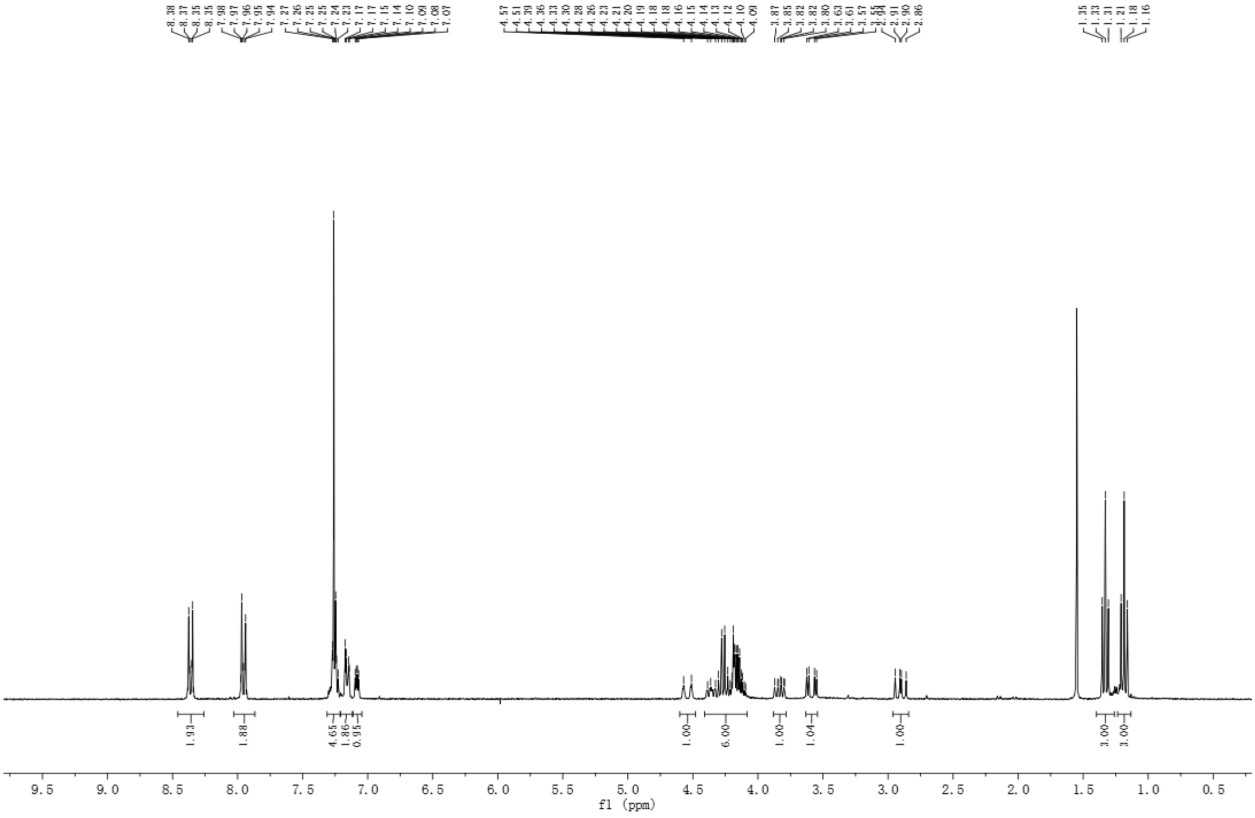

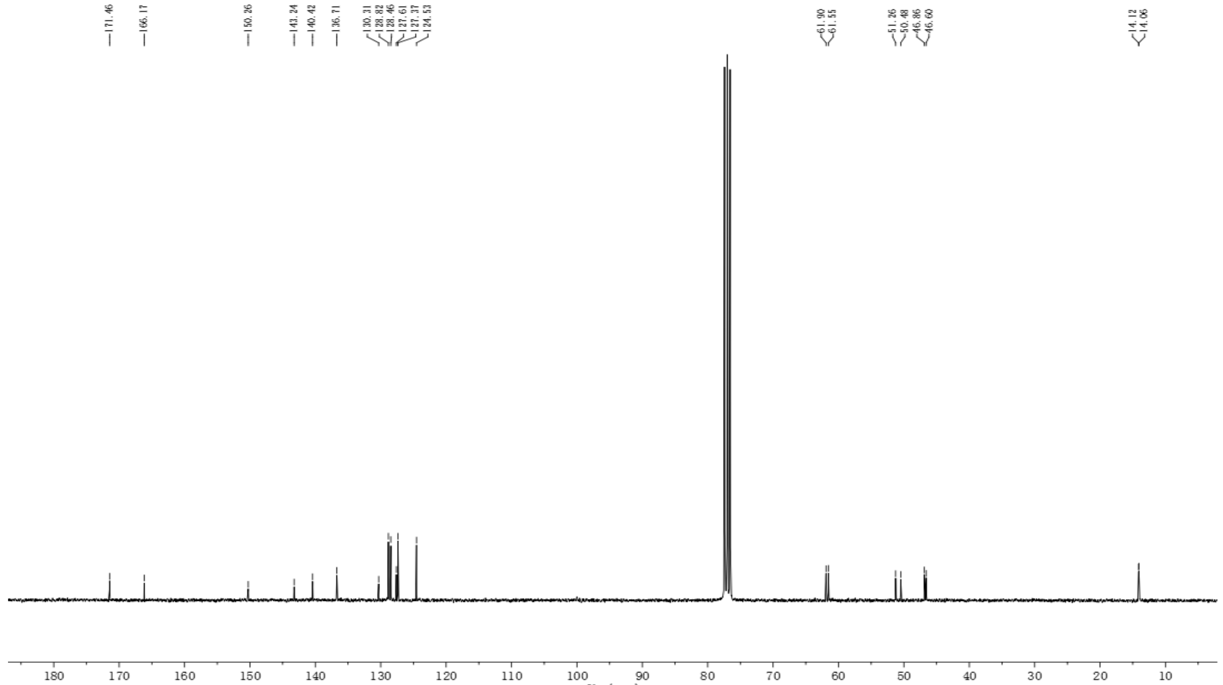

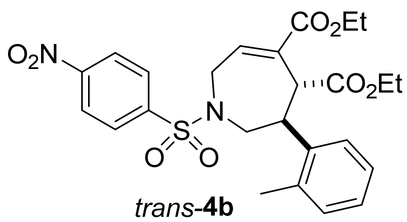

CO2Et
O2N
CO2Et
N
S
O
O

trans-**4b**

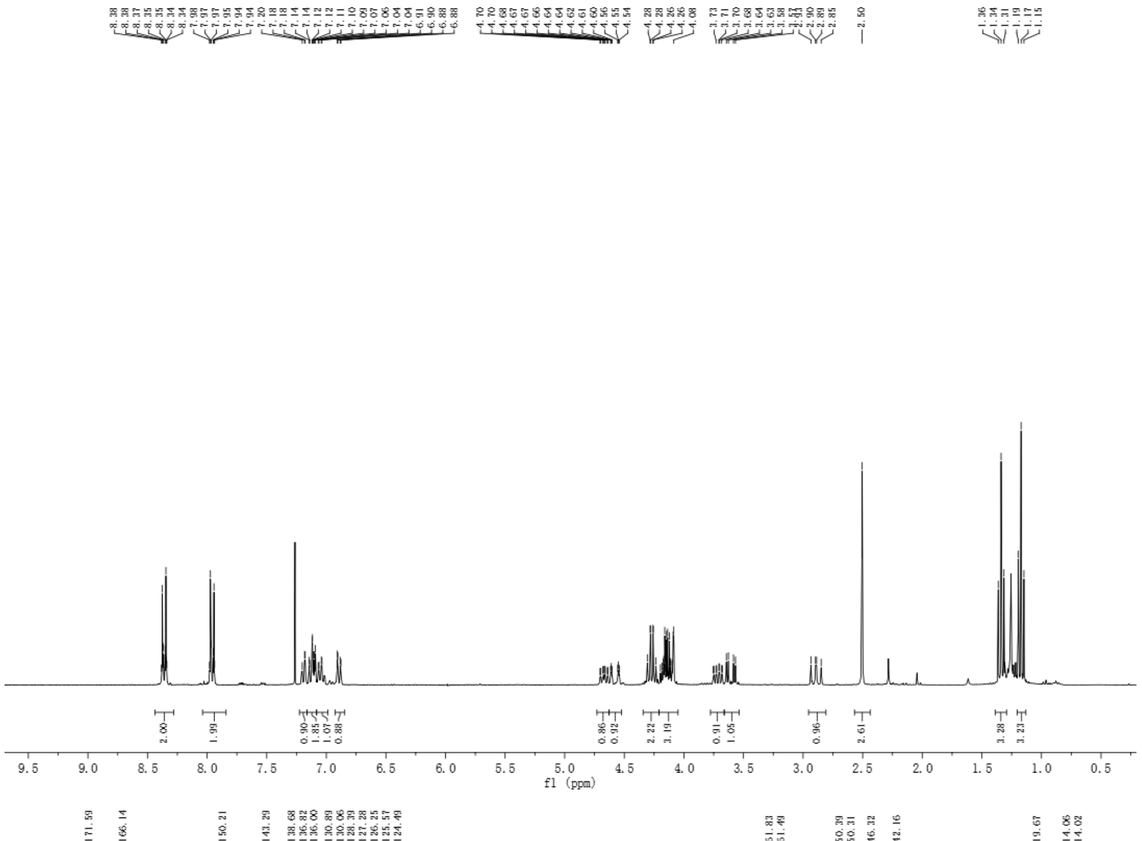

8.38 8.38 8.37 8.35 8.34 8.34 7.98 7.97 7.97 7.95 7.94 7.94 7.20 7.18 7.18 7.14 7.12 7.12 7.11 7.10 7.09 7.07 7.06 7.04 7.04 6.91 6.90 6.88 6.88
4.70 4.70 4.68 4.67 4.66 4.64 4.64 4.62 4.61 4.60 4.56 4.55 4.54 4.28 4.28 4.26 4.26 4.08
3.73 3.71 3.70 3.68 3.64 3.63 3.58 2.90 2.89 2.85 2.50
1.36 1.34 1.31 1.19 1.17 1.15
2.00 1.99 0.90 1.85 1.07 0.88 0.86 0.92 2.22 3.19 0.91 1.05 0.96 2.61 3.28 3.23
9.5 9.0 8.5 8.0 7.5 7.0 6.5 6.0 5.5 5.0 4.5 4.0 3.5 3.0 2.5 2.0 1.5 1.0 0.5
f1 (ppm)

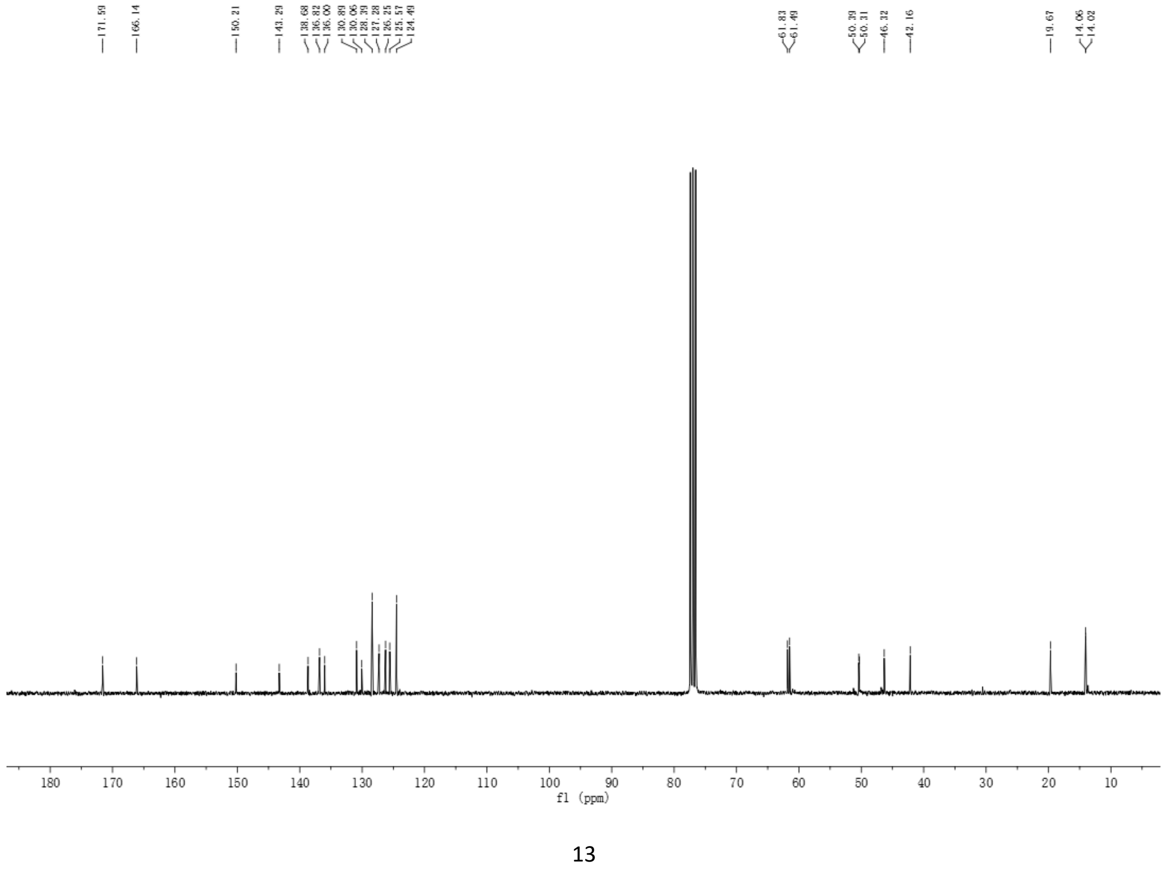

171.59 166.14 150.21 143.29 138.68 136.82 136.00 130.89 130.06 128.39 127.28 126.25 125.57 124.49
61.83 61.49 50.39 50.31 46.32 42.16 19.67 14.06 14.02
180 170 160 150 140 130 120 110 100 90 80 70 60 50 40 30 20 10
f1 (ppm)

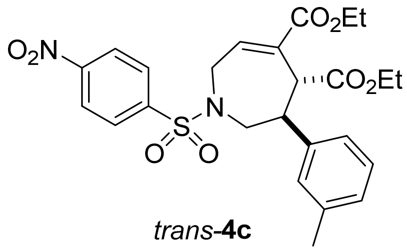

trans-**4c**

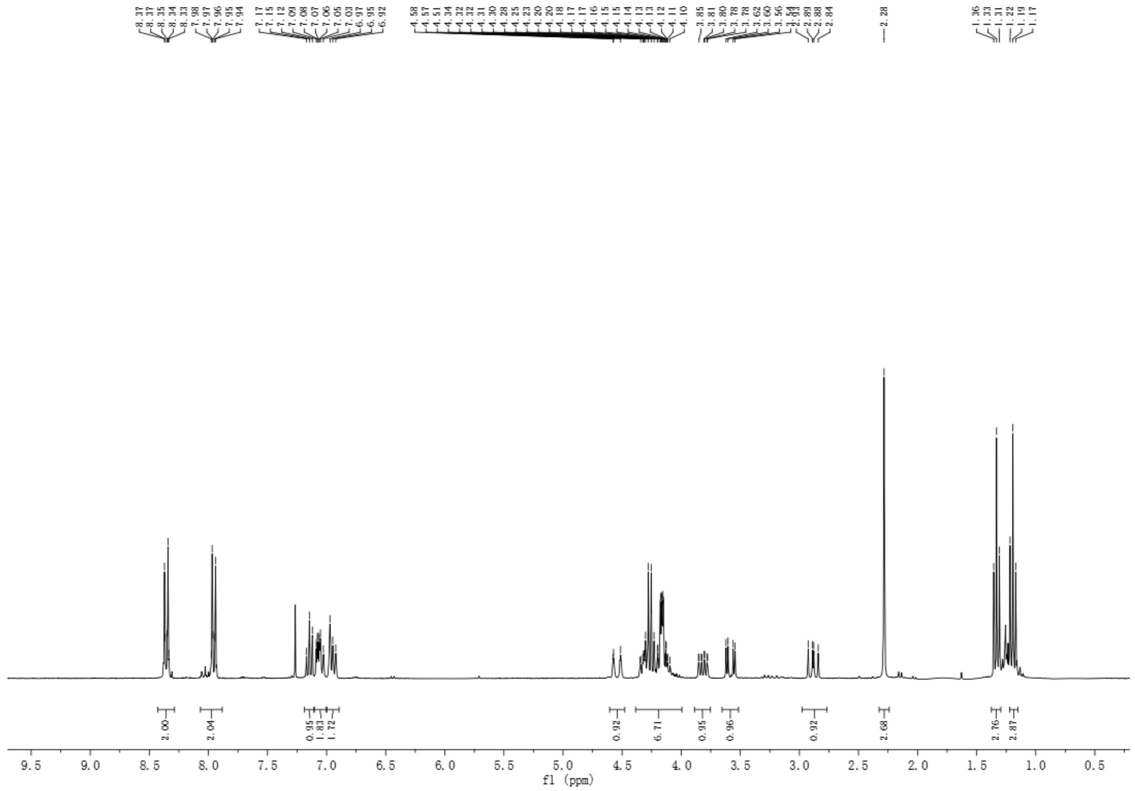

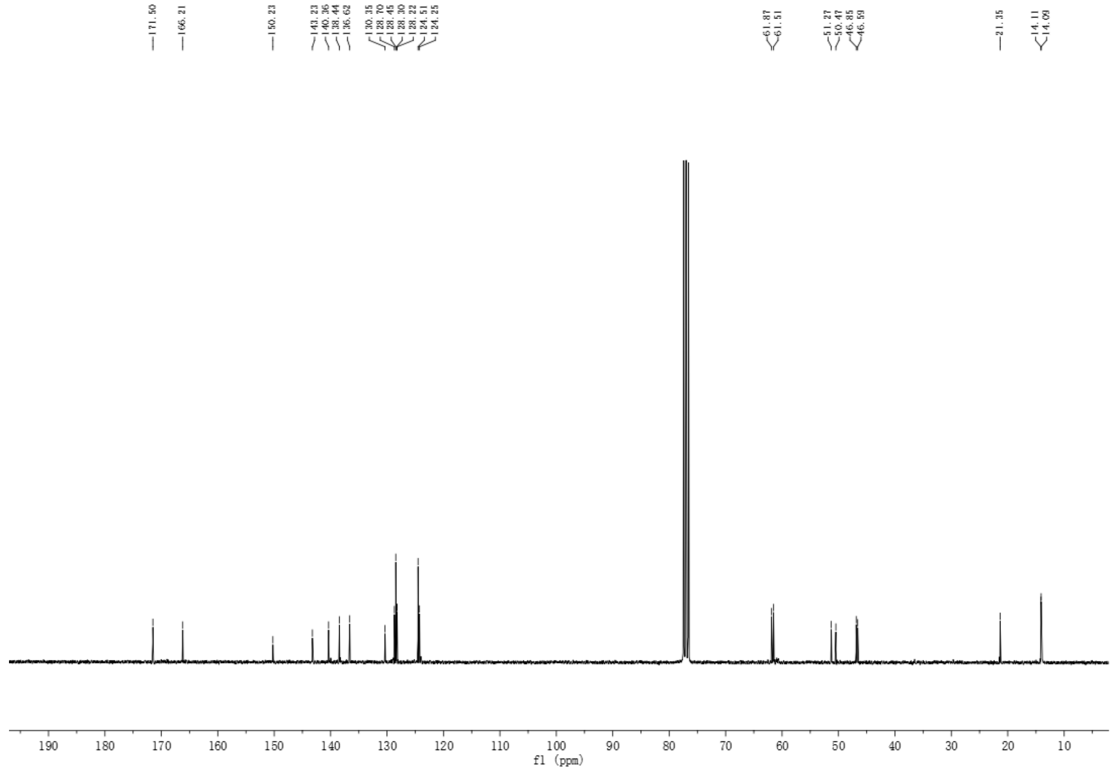

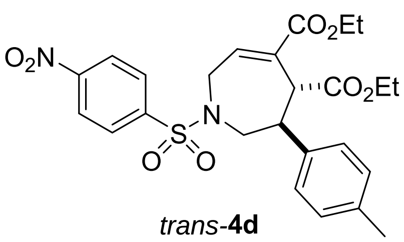

trans-**4d**

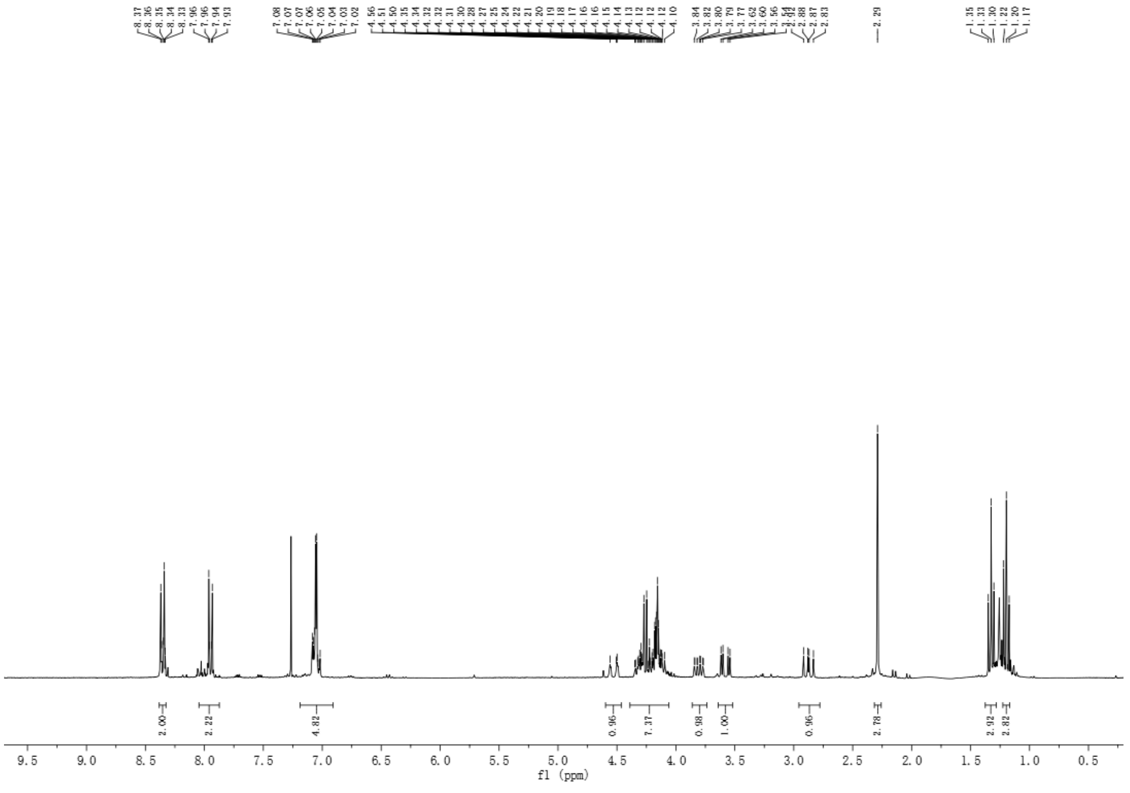
f1 (ppm)

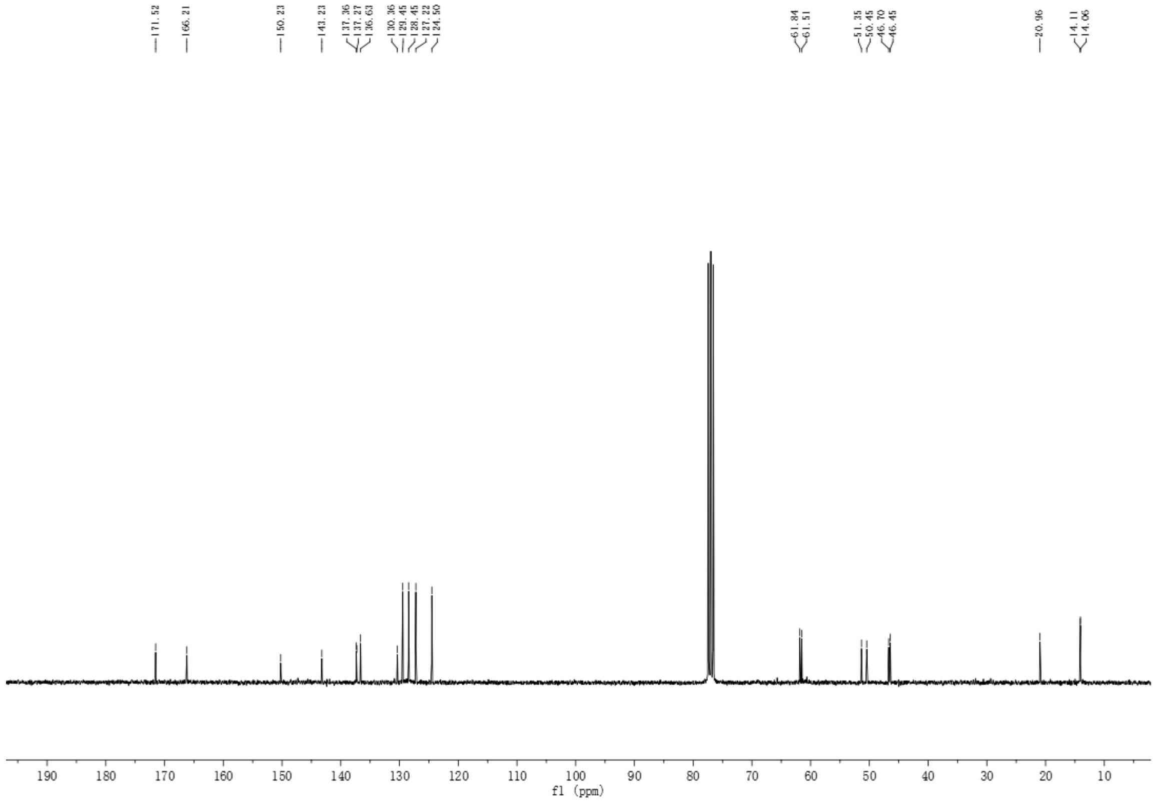
171.52
166.21
150.23
143.23
137.36
137.27
136.63
130.36
129.45
128.45
127.22
124.50
61.84
61.51
51.35
50.45
46.70
46.48
20.96
14.11
14.06
f1 (ppm)

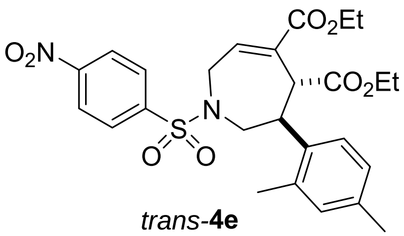
CO2Et
O2N
CO2Et
N
S
O
O

trans-**4e**

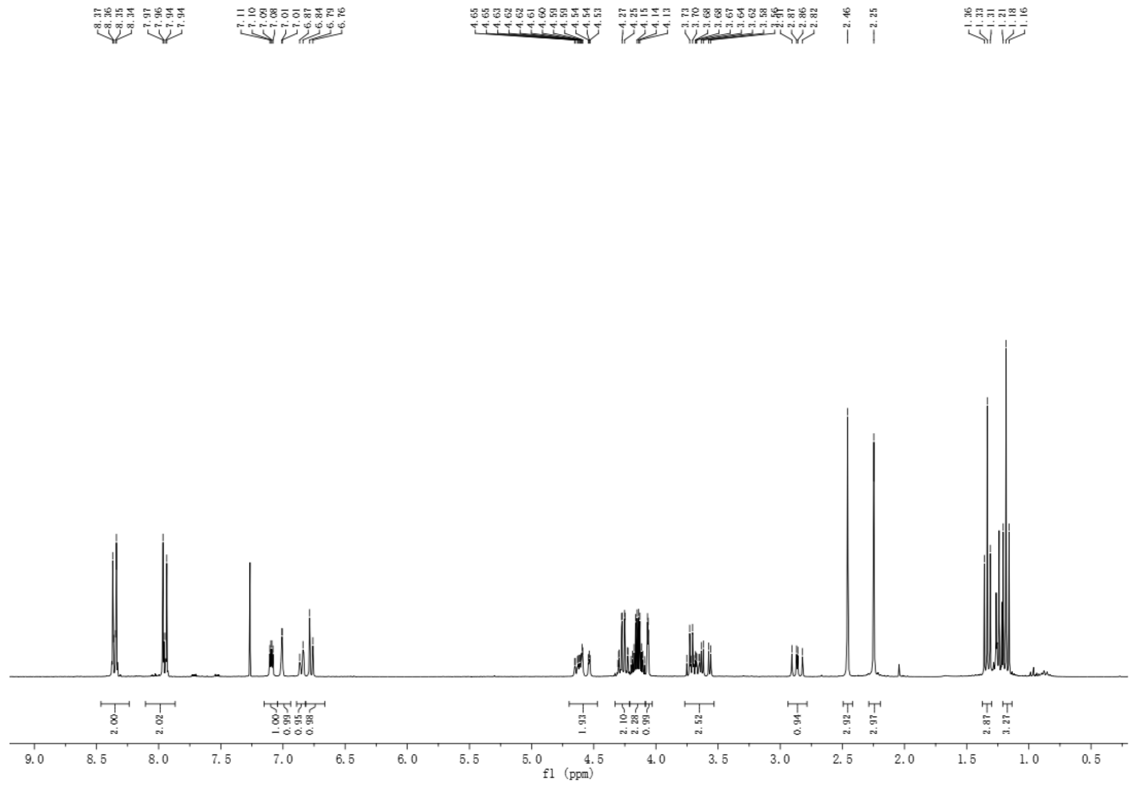
8.37 8.36 8.35 8.34 7.97 7.96 7.94 7.94
7.11 7.10 7.09 7.08 7.01 7.01 6.87 6.84 6.79 6.76
4.65 4.65 4.63 4.62 4.62 4.61 4.60 4.59 4.59 4.54 4.54 4.53
4.27 4.25 4.15 4.14 4.13
3.73 3.70 3.68 3.68 3.67 3.64 3.62 3.58 3.56 2.91 2.87 2.86 2.82
2.46
2.25
1.36 1.33 1.31 1.21 1.18 1.16
2.00 2.02 1.00 0.99 0.95 0.98 1.93 2.10 2.28 0.99 2.52 0.94 2.92 2.97 2.87 3.27
9.0 8.5 8.0 7.5 7.0 6.5 6.0 5.5 5.0 4.5 4.0 3.5 3.0 2.5 2.0 1.5 1.0 0.5
f1 (ppm)

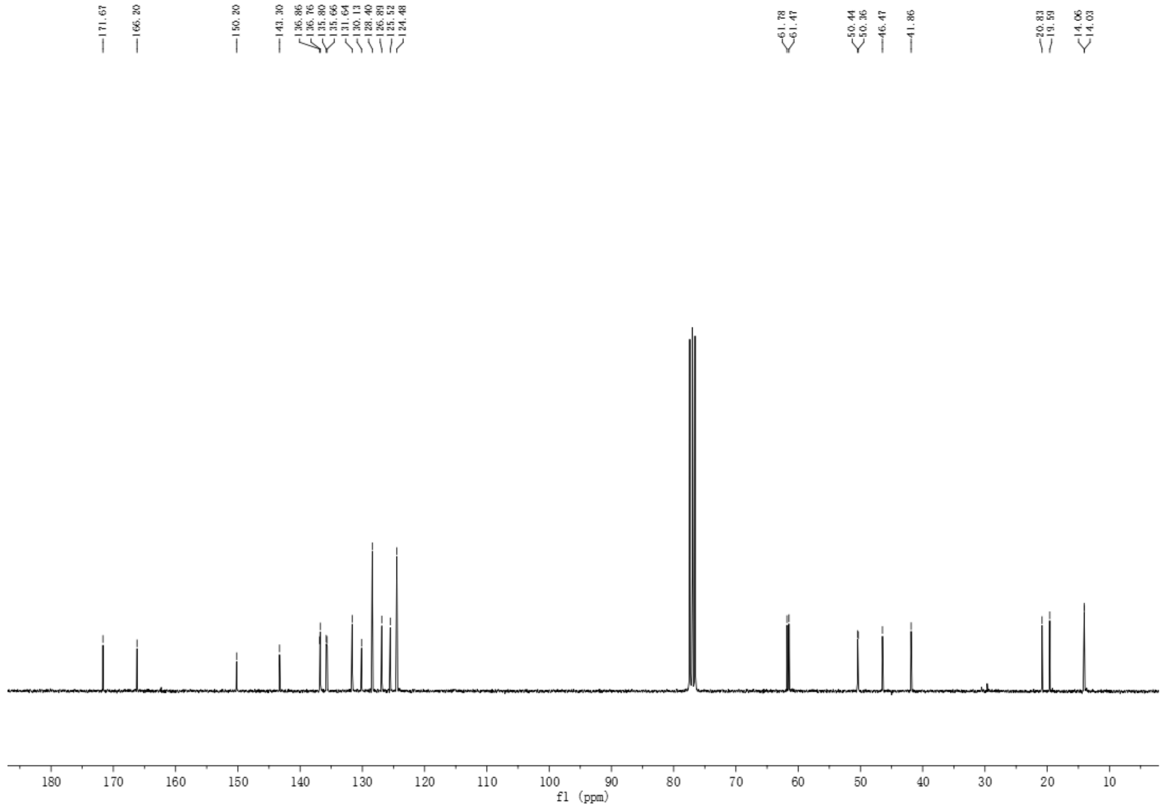
171.67
166.20
150.20
143.30
136.86 136.76 135.80 135.66 131.64 130.13 128.40 126.89 125.52 124.48
61.78 61.47
50.44 50.36
46.47
41.86
20.83 19.59
14.06 14.03
180 170 160 150 140 130 120 110 100 90 80 70 60 50 40 30 20 10
f1 (ppm)

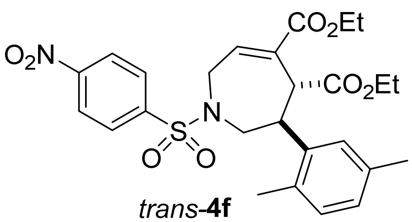
O2N
CO2Et
CO2Et
N
S
O
O

trans-**4f**

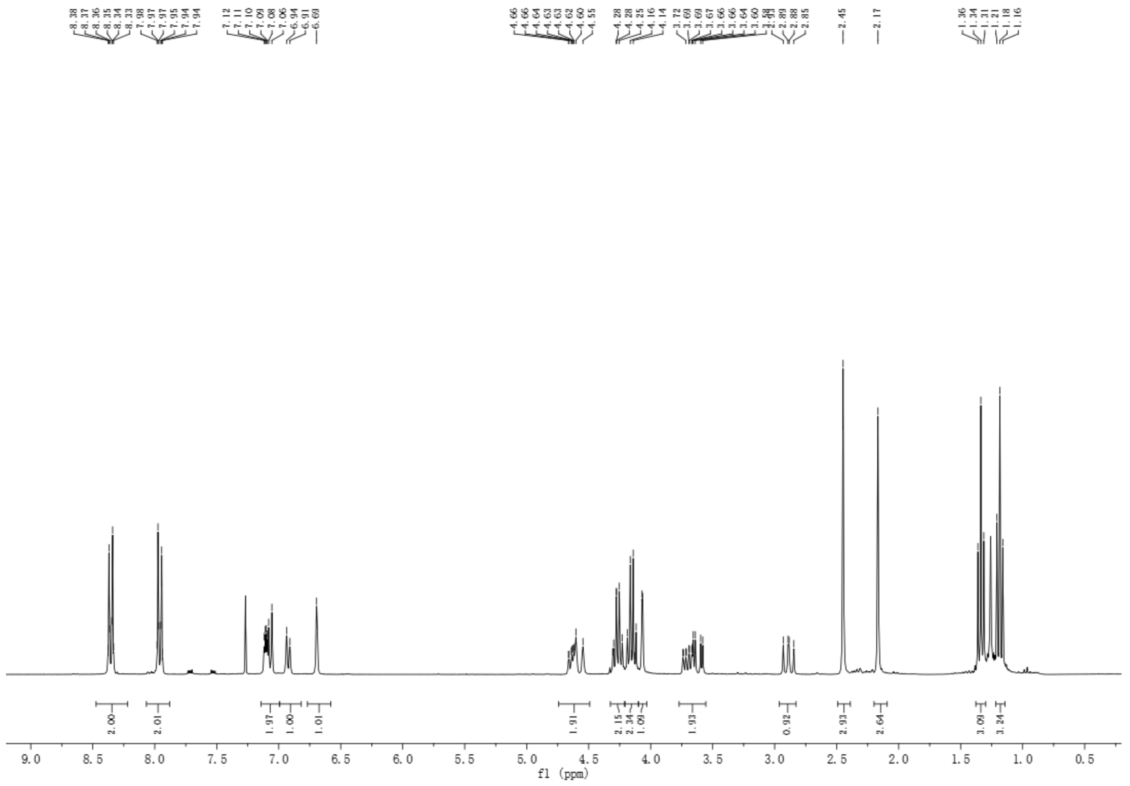
9.0 8.5 8.0 7.5 7.0 6.5 6.0 5.5 5.0 4.5 4.0 3.5 3.0 2.5 2.0 1.5 1.0 0.5
f1 (ppm)

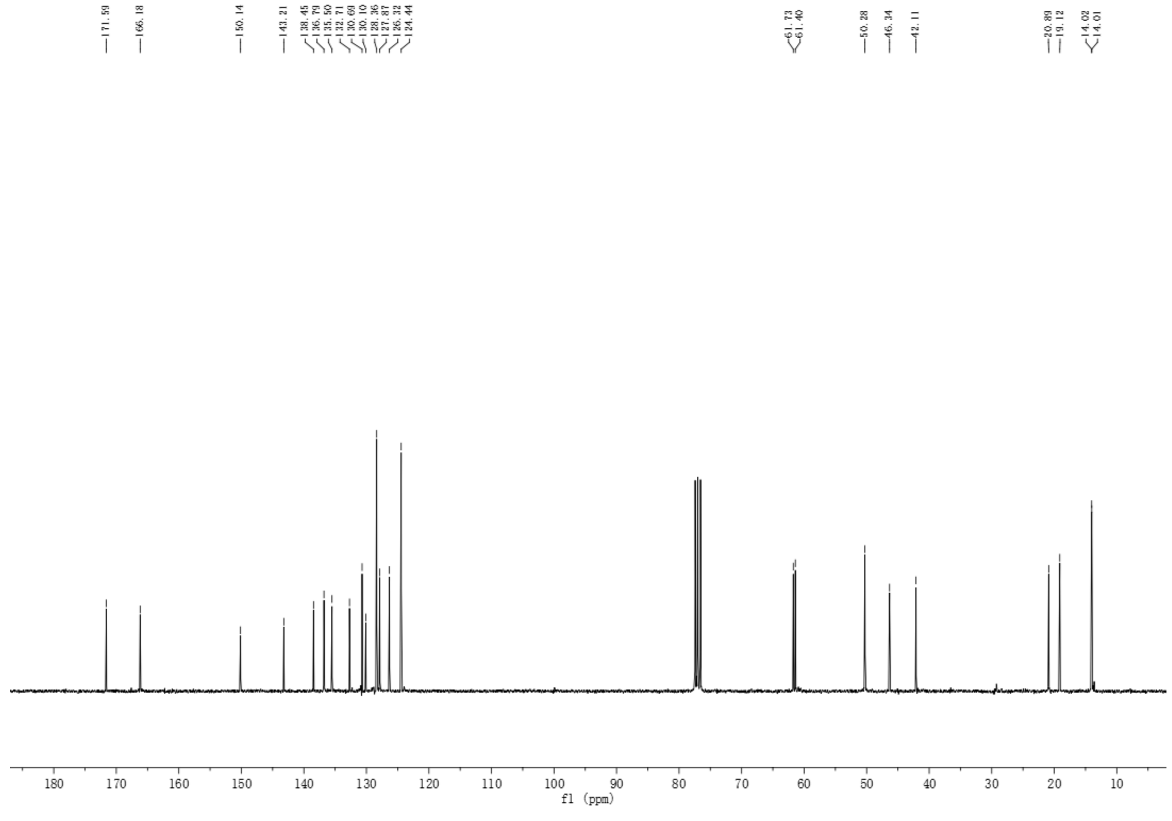
180 170 160 150 140 130 120 110 100 90 80 70 60 50 40 30 20 10
f1 (ppm)

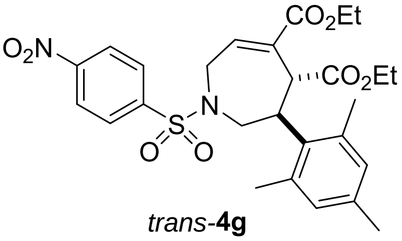

CO2Et
O2N
CO2Et
N
S
O O

trans-**4g**

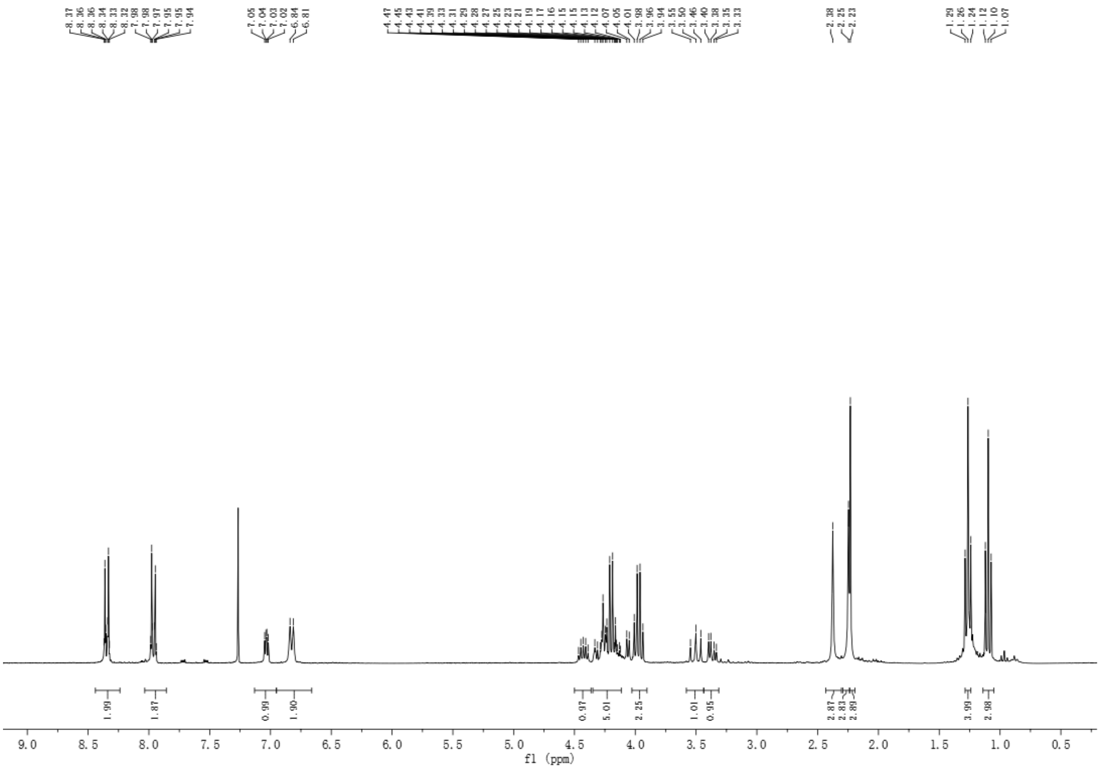

8.37 8.36 8.34 8.33 8.32 7.98 7.98 7.97 7.95 7.95 7.94
7.05 7.04 7.03 7.02 6.84 6.81
4.47 4.45 4.43 4.41 4.39 4.33 4.31 4.29 4.28 4.27 4.25 4.23 4.21 4.19 4.17 4.16 4.15 4.13 4.12 4.07 4.05 4.01 3.98 3.96 3.94 3.55 3.50 3.46 3.40 3.38 3.35 3.33
2.38 2.25 2.23
1.29 1.26 1.24 1.12 1.10 1.07
1.99 1.87 0.99 1.90 0.97 5.01 2.25 1.01 0.95 2.87 2.83 2.89 3.99 2.98
9.0 8.5 8.0 7.5 7.0 6.5 6.0 5.5 5.0 4.5 4.0 3.5 3.0 2.5 2.0 1.5 1.0 0.5
f1 (ppm)

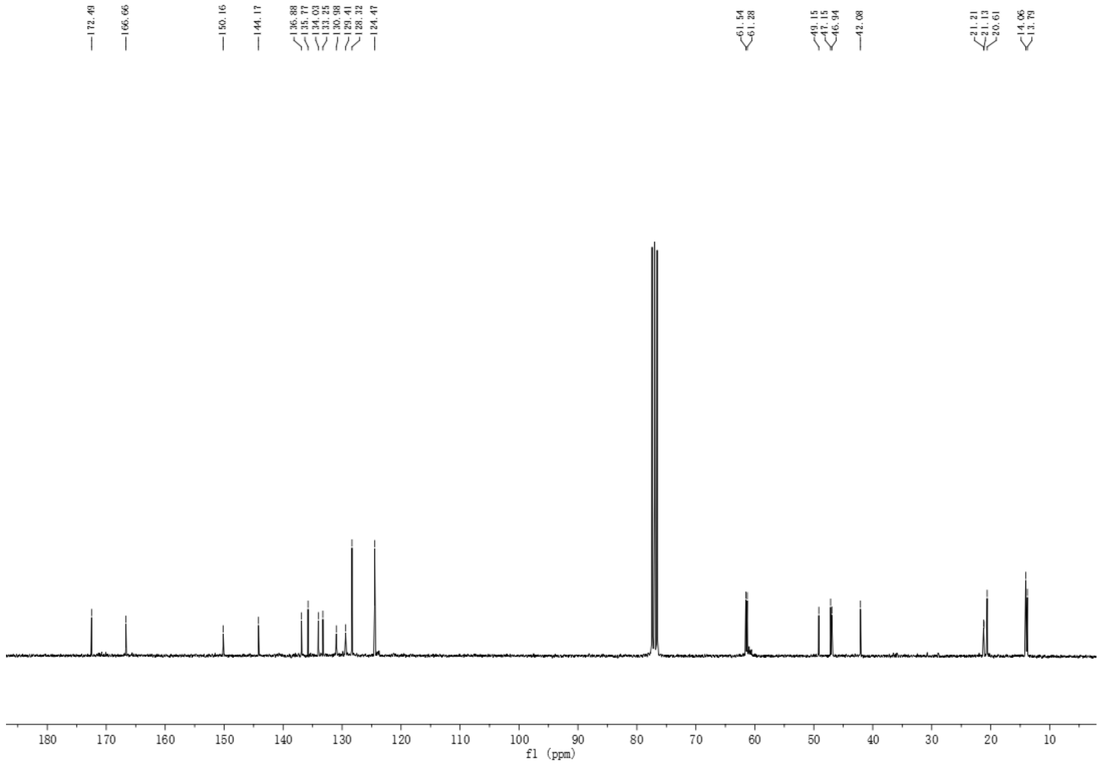

172.49 166.66 150.16 144.17 136.88 135.77 134.03 133.25 130.98 129.41 128.32 124.47
61.54 61.28 49.15 47.15 46.94 42.08 21.21 21.13 20.61 14.06 13.79
180 170 160 150 140 130 120 110 100 90 80 70 60 50 40 30 20 10
f1 (ppm)

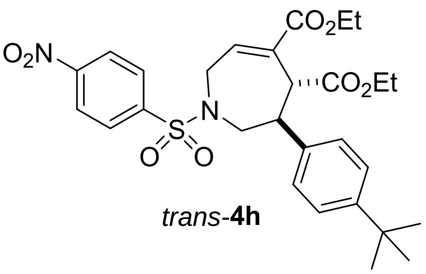

trans-**4h**

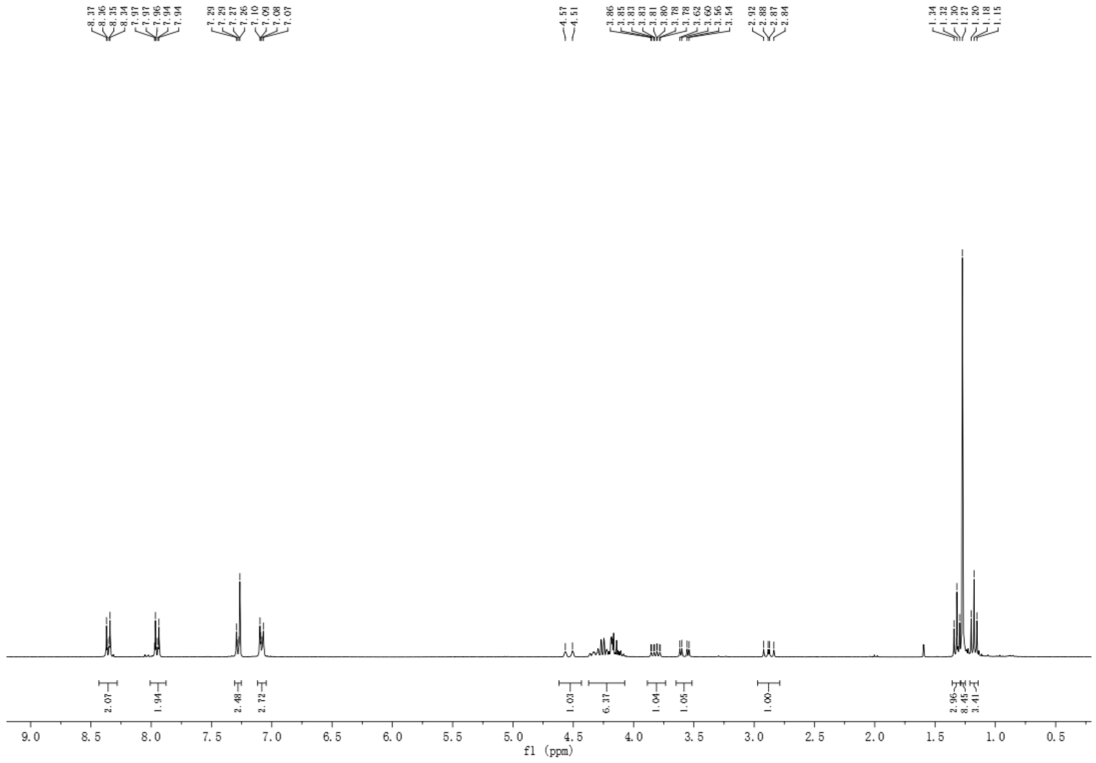

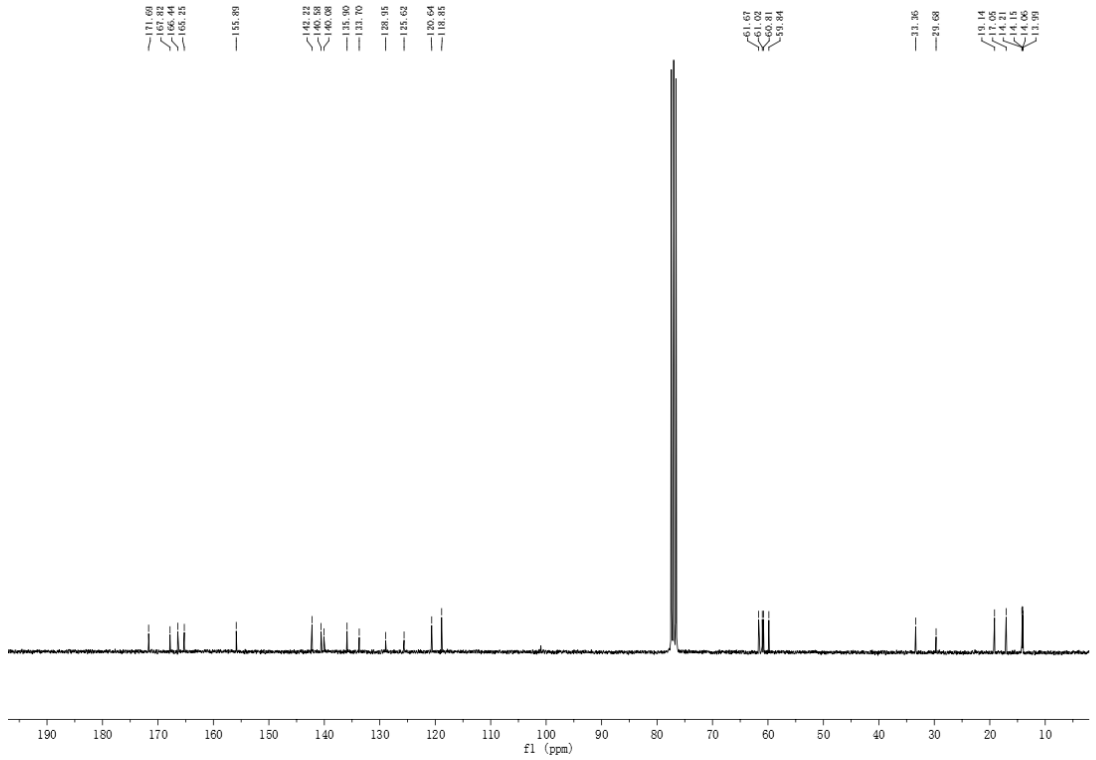

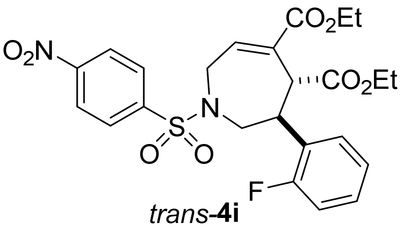

trans-**4i**

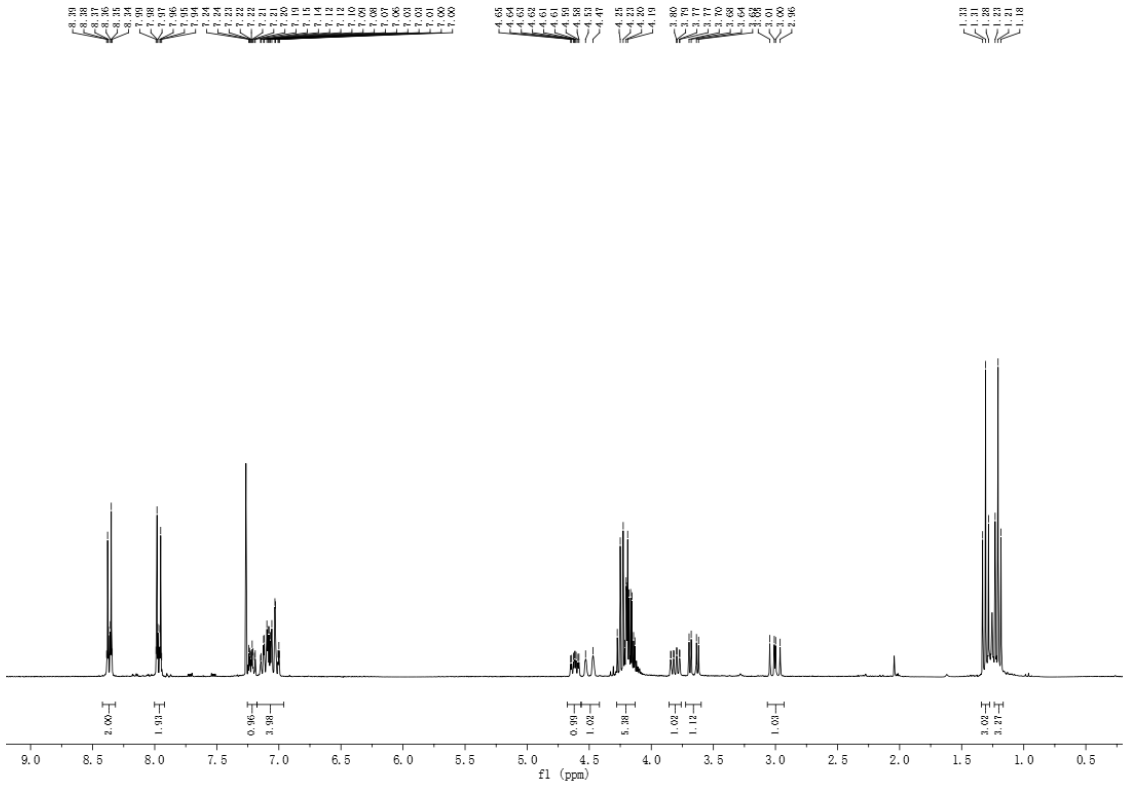

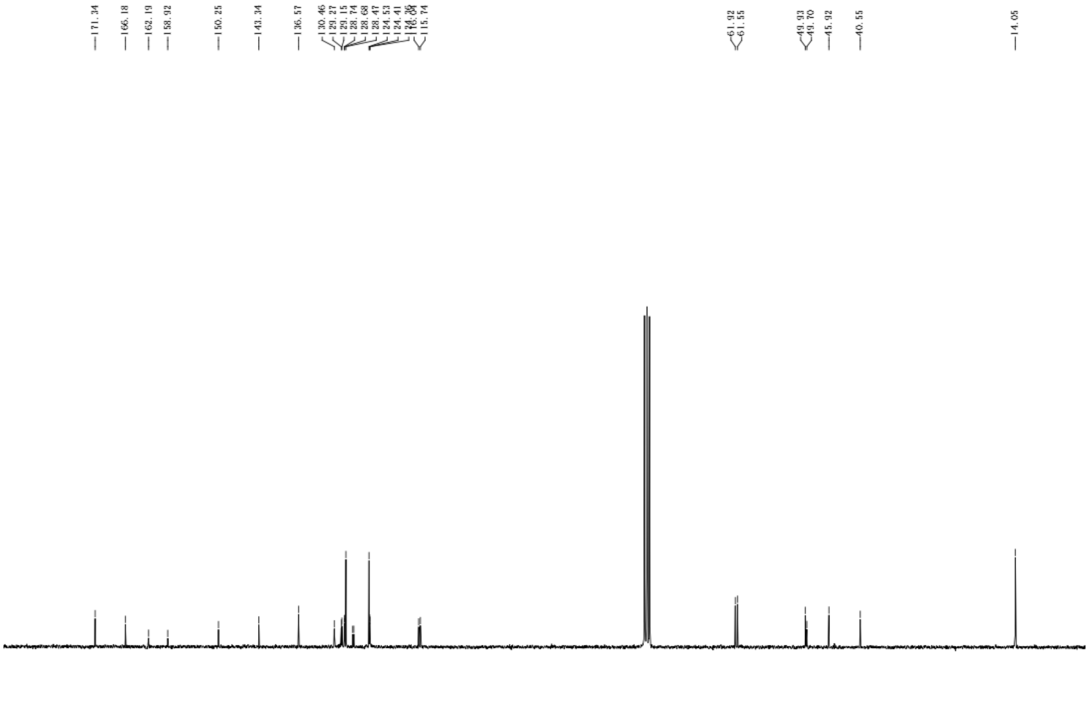

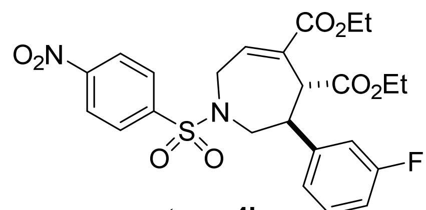

CO_2Et
O_2N
CO_2Et
N
S
O
O
F

trans-**4j**

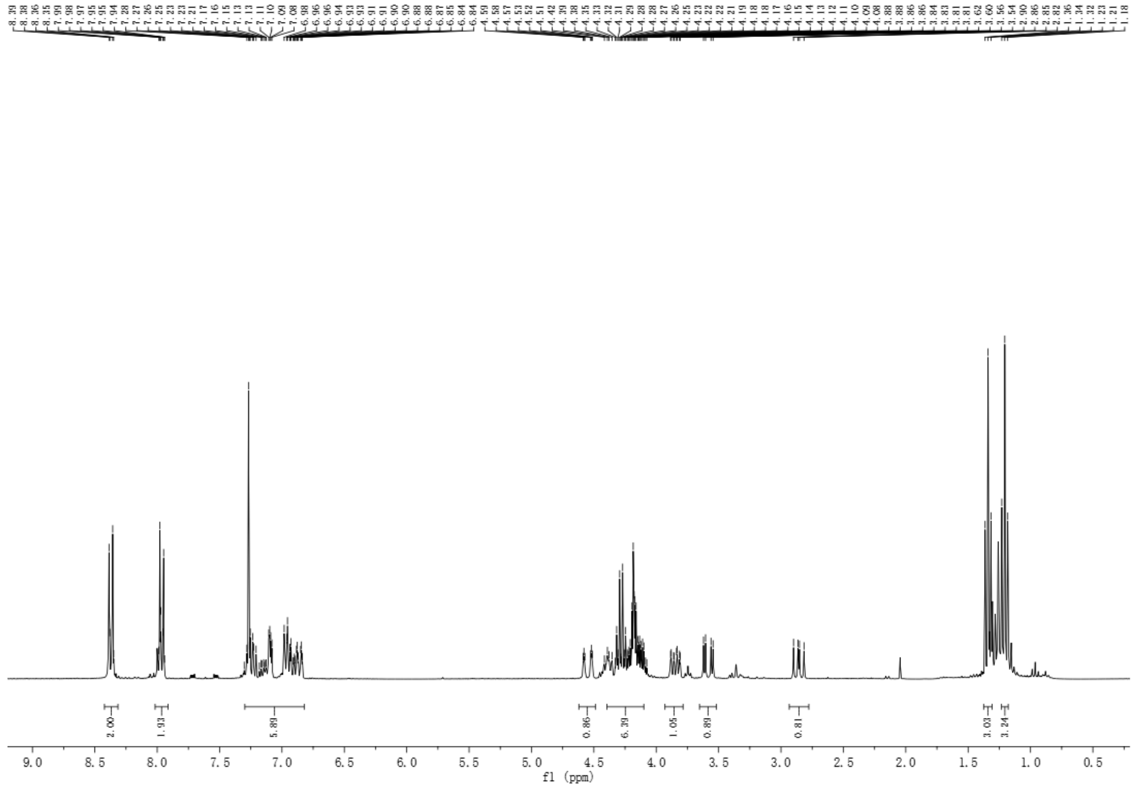

2.00
1.93
5.89
0.86
6.39
1.05
0.89
0.81
3.03
3.24
9.0
8.5
8.0
7.5
7.0
6.5
6.0
5.5
5.0
4.5
4.0
3.5
3.0
2.5
2.0
1.5
1.0
0.5
f1 (ppm)

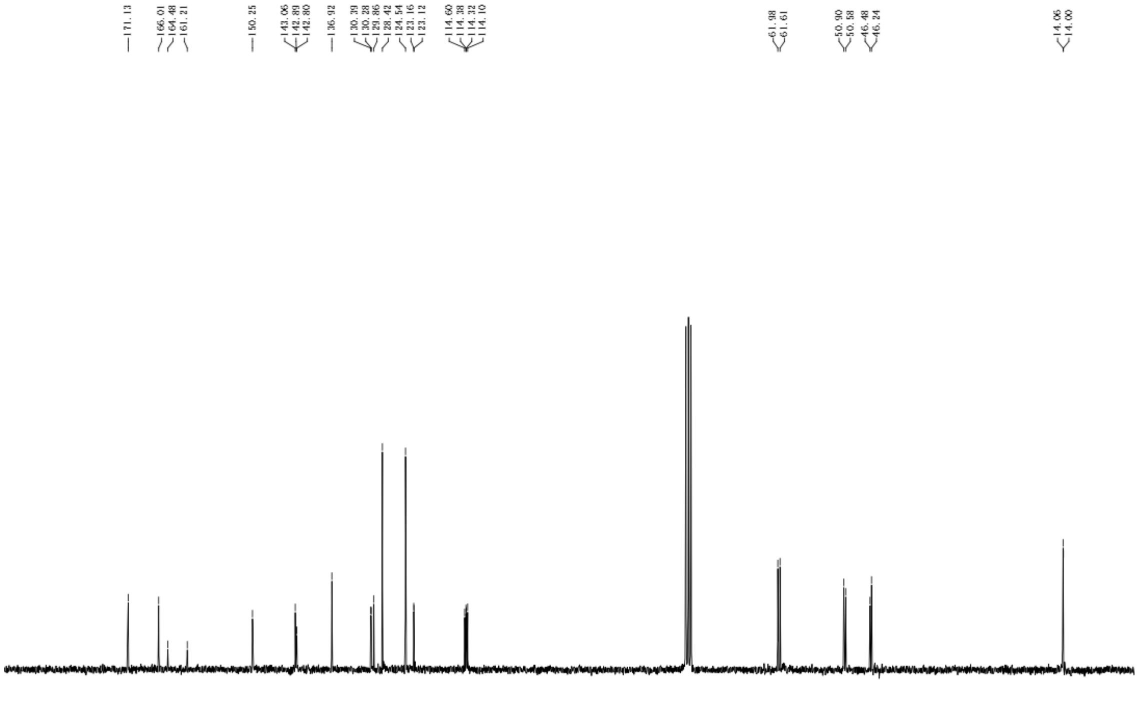

171.13
166.01
164.48
161.21
150.25
143.06
142.89
142.80
136.92
130.39
130.28
129.86
128.42
124.54
123.16
123.12
114.60
114.38
114.32
114.10
61.98
61.61
50.90
50.58
46.48
46.24
14.06
14.00
190
180
170
160
150
140
130
120
110
100
90
80
70
60
50
40
30
20
10
f1 (ppm)

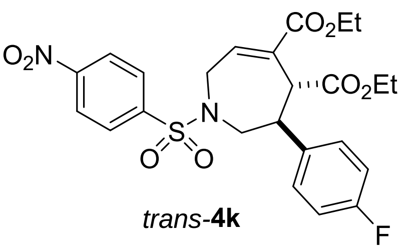

trans-**4k**

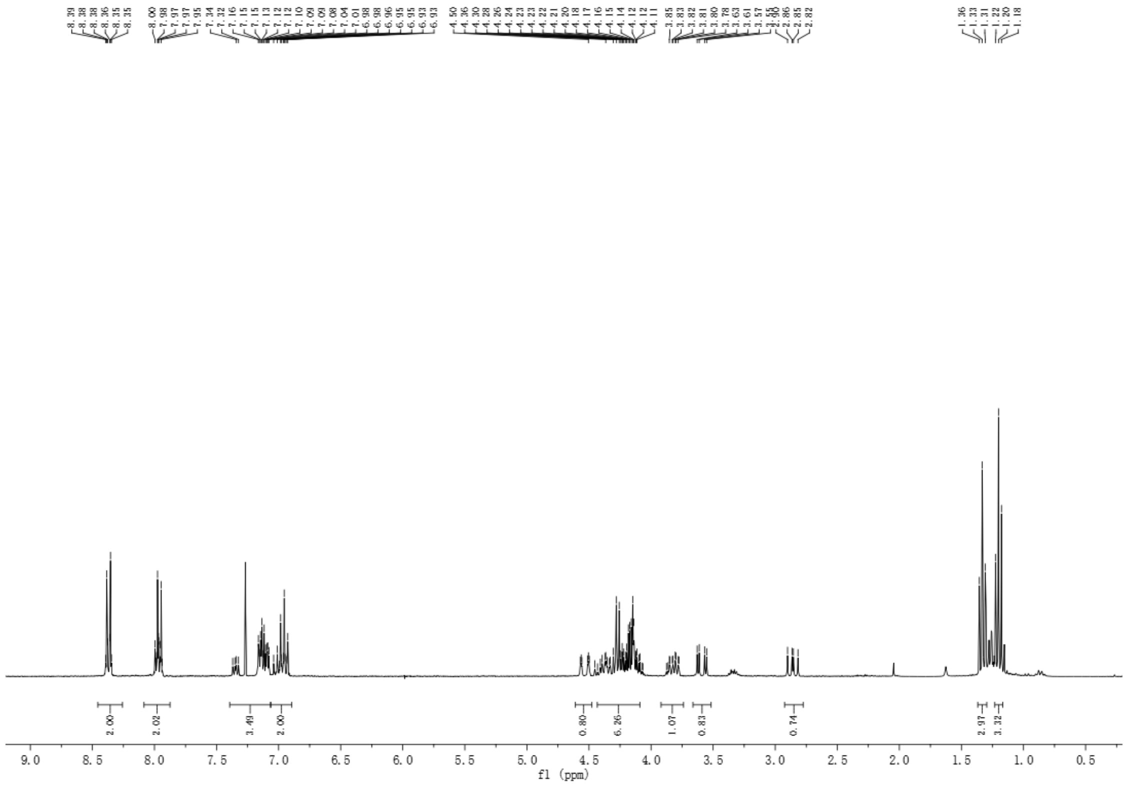

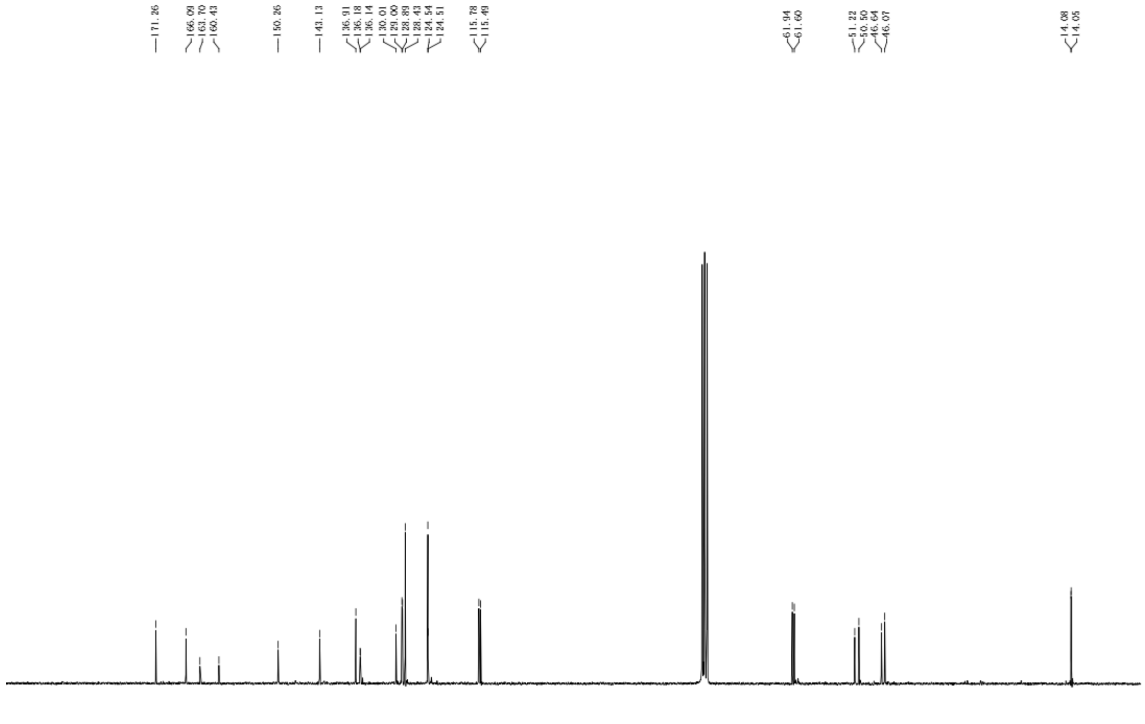

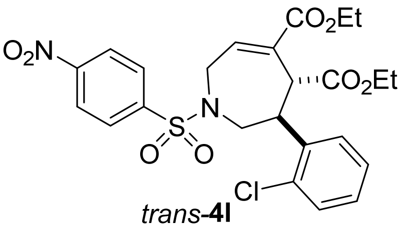

trans-**4l**

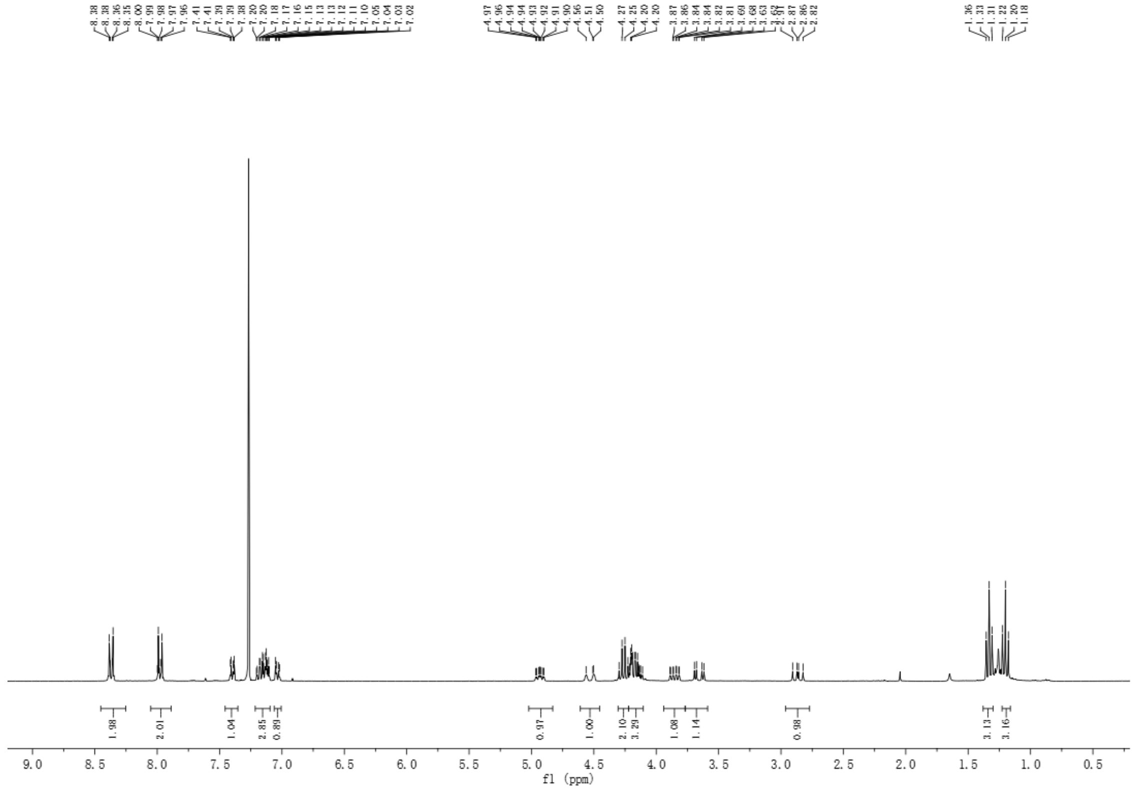

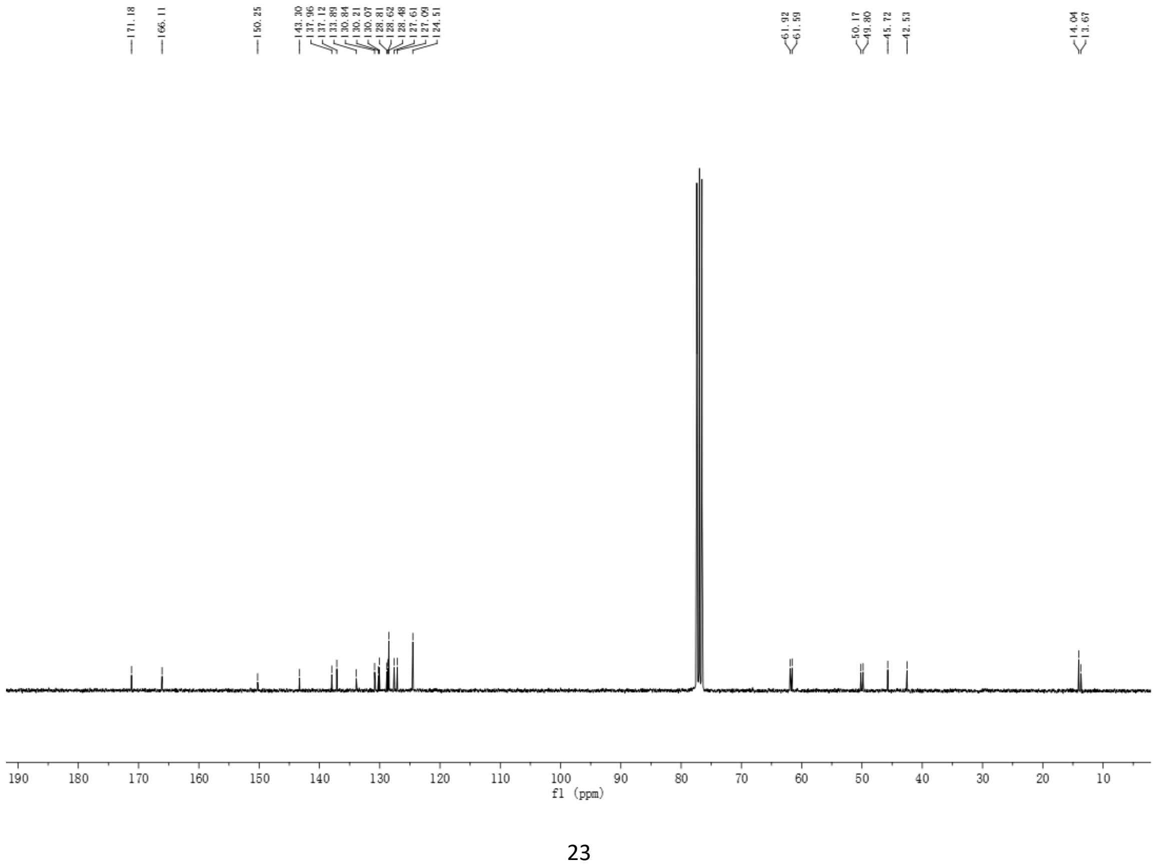

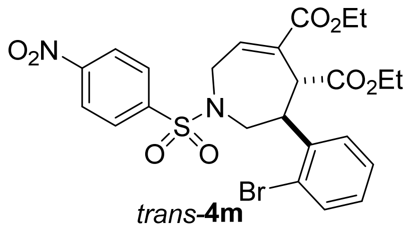

trans-**4m**

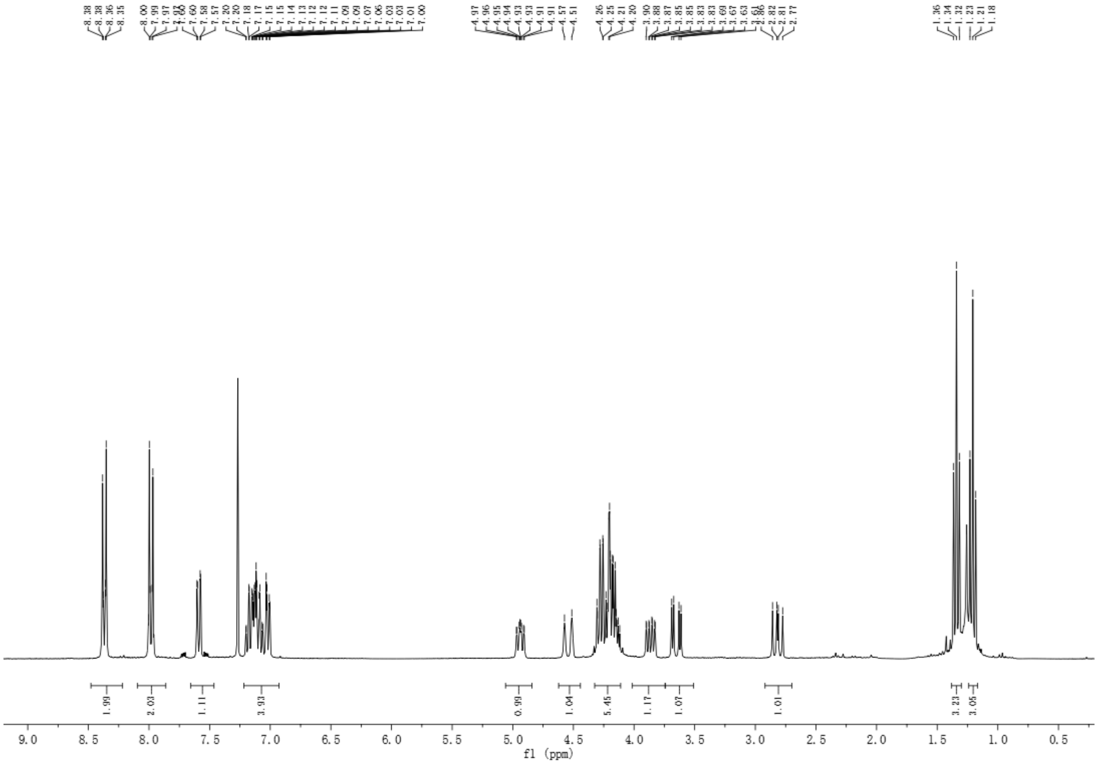

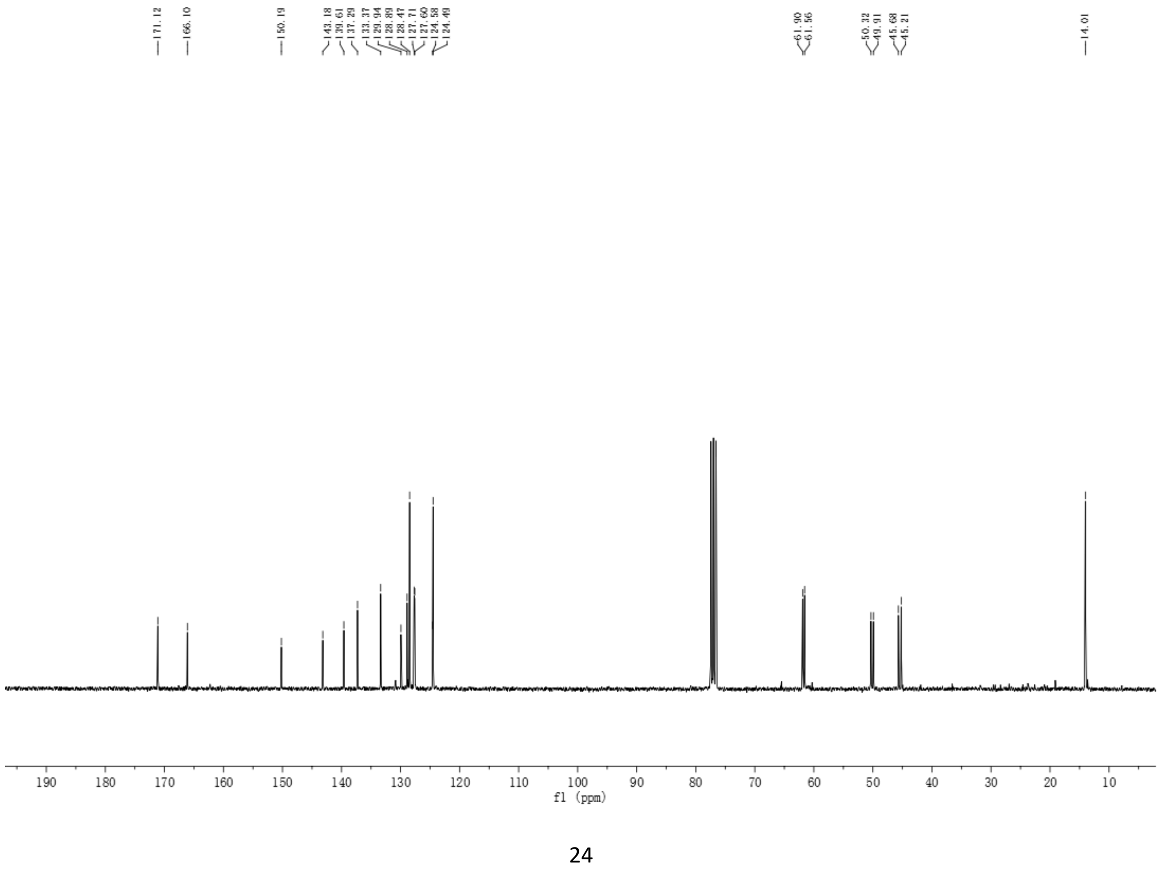

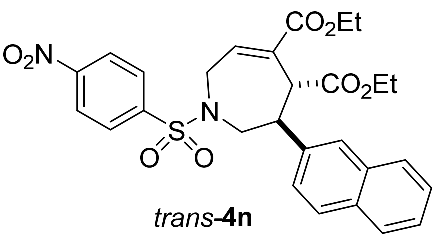

trans-**4n**

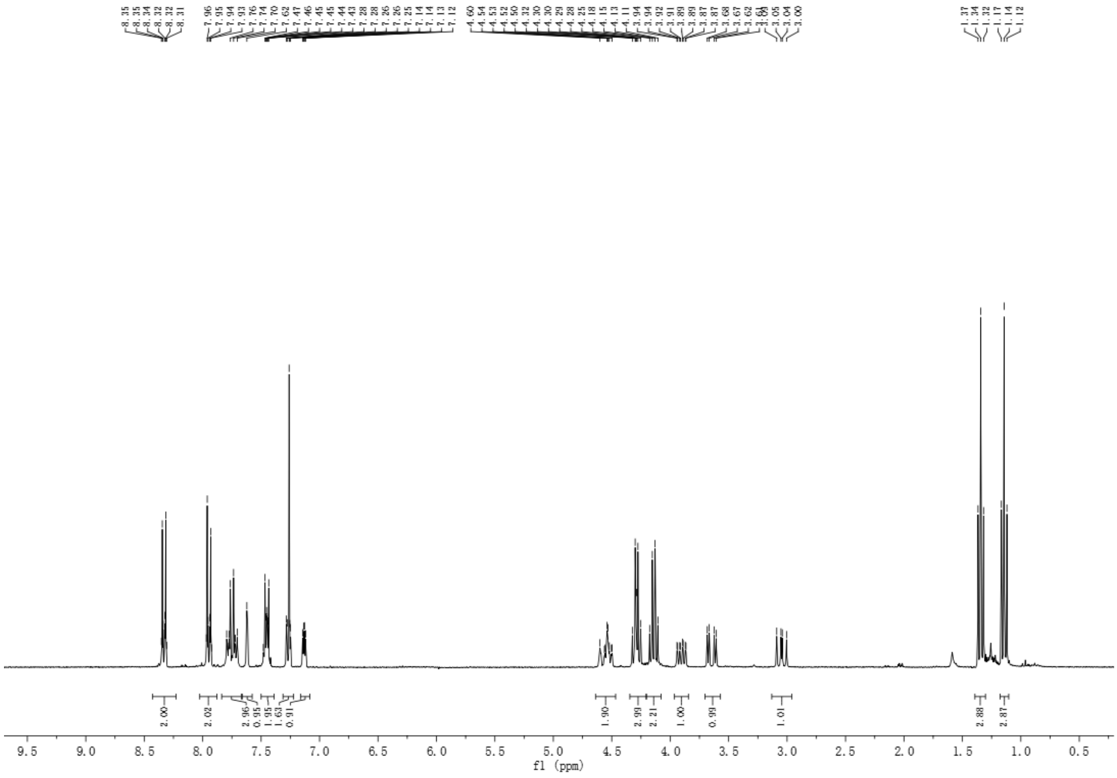

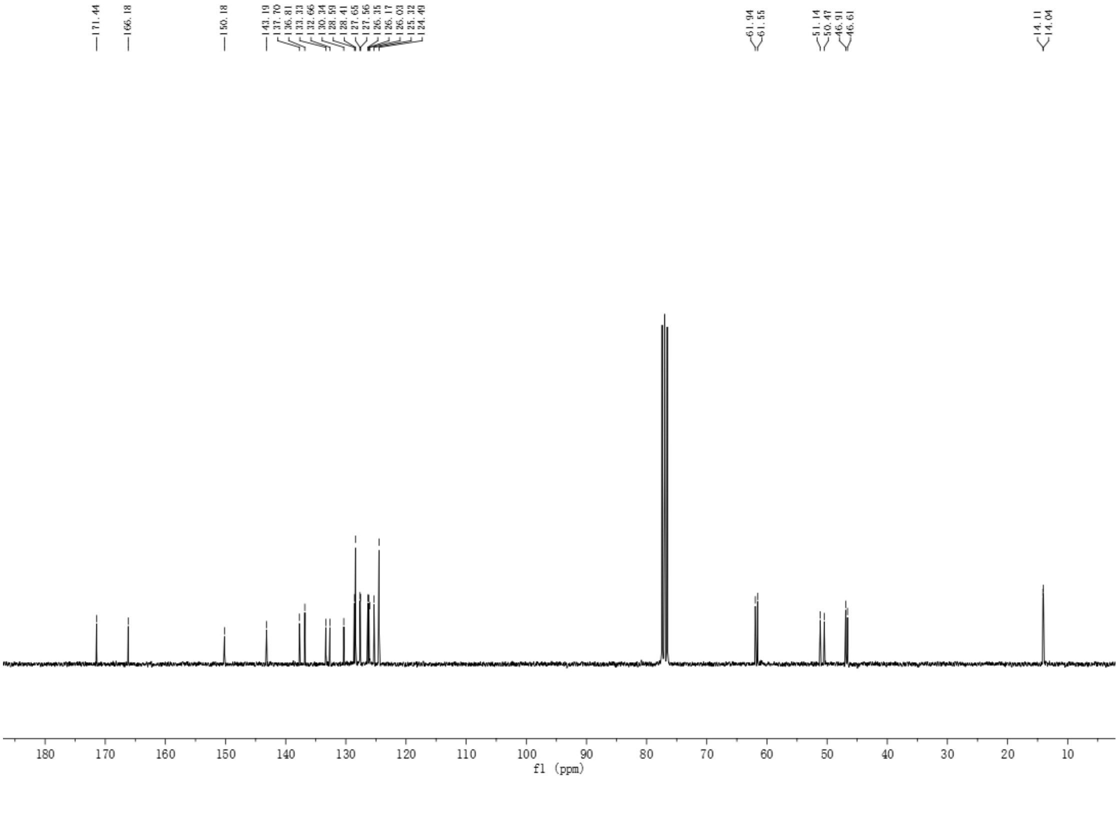

X-Ray Crystallographic Information

Crystallographic data for *trans*-**4a** has been deposited with the Cambridge Crystallographic Data Centre as. These data can be obtained free of charge via www.ccdc.cam. ac.uk/data_request/cif, or by emailing data_request@ccdc.cam.ac.uk, or by contacting The Cambridge Crystallographic Data Centre, 12, Union Road, Cambridge CB2 1EZ, UK; fax: +44 1223 336033.

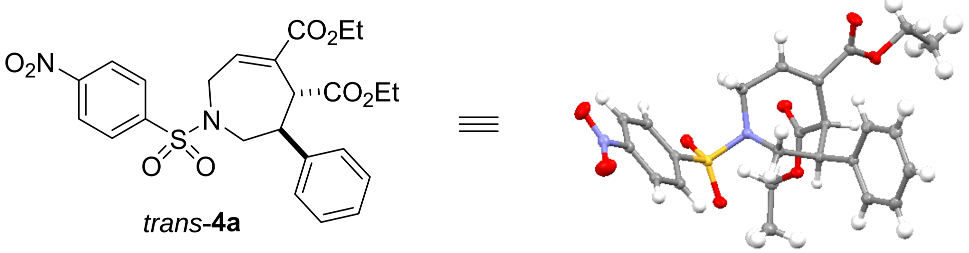

trans-**4a**

Table 1. Crystal data and structure refinement for *trans*-**4a**.

Identification code	*trans*-**4a**	
Empirical formula	$C_{36}H_{28}N_2O_8S_2$	
Formula weight	528.58	
Space group	P^{-1}	
Unit cell dimensions	a = 10.136(3) Å	alpha= 104.454 (3)°.
	b = 10.925(3) Å	beta= 109.130(3)°.
	c = 11.309 (2) Å	gamma = 91.112(3)°.
Volume	1138.67(4) $Å^3$	
Z	2	
Z’	0	

Table 2. Bond lengths [Å] and angles [°] for *trans*-**4a**.

S1-O3	1.430(1)
S1-O4	1.429(1)
S1-N2	1.631(1) 1
S1-C6	1.772(2) 1
O1-N1	1.213(2) 1
O2-N1	1.222(2) 1
O5-C19	1.205(1) 2

O6-C19	1.338(2) 1
O6-C20	1.455(2) 1
O7-C22	1.211(2) 2
O8-C22	1.342(2) 1
O8-C23	1.454(2) 1
N1-C3	1.479(2) 1
N2-C7	1.470(2) 1
N2-C12	1.469(2) 1
C1-H1	0.99(2) 1
C1-C2	1.391(3)
C1-C6	1.388(2) 1
C2-H2	0.93(2) 1
C2-C3	1.384(2)
C3-C4	1.380(2)
C4-H4	0.98(2) 1
C4-C5	1.391(2)
C5-H5	0.92(2) 1
C5-C6	1.391(2) 1
C7-H7A	0.96(2) 1
C7-H7B	1.494(2) 1
C8-H8	0.98(2) 1
C8-C9	1.332(2)
C9-C10	1.520(2) 1
C9-C22	1.489(2)
C10-H10	0.98(2) 1
C10-C11	1.553(2) 1

C10-C19	1.532(2) 1
C11-H11	0.98(2) 1
C11-C12	1.543(2) 1
C11-C13	1.513(2) 1
C12-H12A	0.99(2) 1
C12-H12B	0.99(2) 1
C13-C14	1.390(2)
C13-C18	1.396(2)
C14-H14	0.96(2) 1
C14-C15	1.389(2)
C15-H15	0.95(2) 1
C15-C16	1.384(2)
C16-H16	0.91(2) 1
C16-C17	1.384(2)
C17-H17	0.96(2) 1
C17-C18	1.389(2)
C18-H18	0.95(1) 1
C20-H20A	0.96(2) 1
C20-H20B	1.00(2) 1
C20-C21	1.499(2) 1
C21-H21A	0.99(2) 1
C21-H21B	0.98(2) 1
C21-H21C	0.97(2) 1
C23-H23A	0.97(2) 1
C23-H23B	0.93(2) 1
C23-C24	1.502(3) 1

C24-H24A	0.99(2) 1
C24-H24B	0.97(2) 1
C24-H24C	0.97(2) 1
O3-S1-O4	120.51(6)
O3-S1-N2	106.93(6)
O3-S1-C6	108.28(7)
O4-S1-N2	106.93(6)
O4-S1-C6	107.76(7)
N2-S1-C6	105.48(7)
C19-O6-C20	115.2(1)
C22-O8-C23	115.8(1)
O1-N1-O2	124.2(1)
O1-N1-C3	118.1(1)
O2-N1-C3	117.7(1)
S1-N2-C7	117.6(1)
S1-N2-C12	118.5(1)
C7 N2 C12	114.3(1)
H1-C1-C2	121(1)
H1-C1-C6	120(1)
C2-C1-C6	119.5(1)
C1-C2-H2	120(1)
C1-C2-C3	117.7(1)
H2-C2-C3	122(1)
N1-C3-C2	117.8(1)
N1-C3-C4	118.5(1)
C2-C3-C4	123.6(1)

C3-C4-H4	121(1)
C3-C4-C5	118.3(1)
H4-C4-C5	120(1)
C4-C5-H5	120(1)
C4-C5-C6	119.0(1)
H5-C5-C6	121(1)
S1-C6-C1	118.9(1)
S1-C6-C5	119.3(1)
C1-C6-C5	121.8(1)
N2-C7-H7A	111(1)
N2-C7-H7B	110(1)
N2-C7-C8	112.4(1)
H7A-C7-H7B	107(2)
H7A-C7-C8	107(1)
H7B-C7-C8	108(1)
C7-C8-H8	115(1)
C7-C8-C9	128.3(1)
H8-C8-C9	116(1)
C8-C9-C10	125.2(1)
C8-C9-C22	115.4(1)
C10-C9-C22	119.1(1)
C9-C10-H10	107(1)
C9-C10-C11	114.3(1)
C9-C10-C19	109.0(1)
H10-C10-C11	107(1)
H10-C10-C19	108(1)

C11-C10-C19	112.4(1)
C10-C11-H11	107(1)
C10-C11-C12	111.1(1)
C10-C11-C13	115.9(1)
H11-C11-C12	106(1)
H11-C11-C13	108(1)
C12-C11-C13	108.4(1)
N2-C12-C11	110.8(1)
N2-C12-H12A	107(1)
N2-C12-H12B	111(1)
C11-C12-H12A	112(1)
C11-C12-H12B	109(1)
H12A-C12-H12B	106(2)
C11-C13-C14	119.4(1)
C11-C13-C18	122.5(1)
C14-C13-C18	117.8(1)
C13-C14-H14	119(1)
C13-C14-C15	121.3(1)
H14 -C14-C15	120(1)
C14-C15-H15	120(1)
C14-C15-C16	120.3(2)
H15-C15-C16	120(1)
C15-C16-H16	118(1)
C15-C16-C17	119.2(1)
H16-C16-C17	122(1)
C16-C17-H17	120(1)

C16-C17-C18	120.4(1)
H17-C17-C18	120(1)
C13-C18-C17	121.1(1)
C13-C18-H18	120(1)
C17-C18-H18	119(1)
O5-C19-O6	123.9(1)
O5-C19-C10	124.3(1)
O6-C19-C10	111.8(1)
O6-C20-H20A	108(1)
O6-C20-H20B	107(1)
O6-C20-C21	107.1(1)
H20A-C20-H20B	111(2)
H20A-C20-C21	112(1)
H20B-C20-C21	111(1)
C20-C21-H21A	111(1)
C20-C21-H21B	110(1)
C20-C21-H21C	111(1)
H21A-C21-H21B	108(2)
H21A-C21-H21C	111(2)
H21B-C21-H21C	106(2)
O7-C22-O8	123.3(1)
O7-C22-C9	125.1(1)
O8-C22-C9	111.5(1)
O8-C23-H23A	107(1)
O8-C23-H23B	106(1)
O8-C23-C24	107.7(1)

H23A-C23-H23B	113(2)
H23A C23 C24	111(1)
H23B C23 C24	112(1)
C23-C24 -H24A	110(1)
C23-C24-H24B	111(1)
C23-C24-H24C	107(1)
H24A-C24-H24B	109(2)
H24A-C24-H24C	109(2)
H24B-C24-H24C	111(2)

Table 3. Torsion angles [°] for *trans*-**4a**.

O3-S1-N2-C7	178.6(1)
O3-S1-N2-C12	-37.0(1)
O4-S1-N2-C7	48.3(1)
O4-S1-N2-C12	-167.3(1)
C6-S1-N2-C7	-66.3(1)
C6-S1-N2-C12	78.1(1)
O3-S1-C6-C1	25.8(1)
O3-S1-C6-C5	-156.6(1)
O4-S1-C6-C1	157.6(1)
O4-S1-C6-C5	-24.8(1)
N2-S1-C6-C1	-88.4(1)
N2-S1-C6-C5	89.2(1)
C2-O6-C19-O5	-5.3(2)
C20-O6-C19-C10	177.2(1)
C19-O6-C20-C21	-174.9(1)
C23-O8-C22-O7	-0.7(2)

C23-O8-C22-C9	177.5(1)
C22-O8-C23-C24	177.8(1)
O1-N1-C3-C2	-6.2(2)
O1-N1-C3-C4	172.9(1)
O2-N1-C3-C2	173.4(1)
O2-N1-C3-C4	-7.4(2)
S1-N2-C7-C8	-161.3(1)
C12-N2-C7-C8	52.9(2)
S1-N2-C12-C11	116.2(1)
C7-N2-C12-C11	-98.2(1)
H1-C1-C2-H2	-0(2)
H1-C1-C2-C3	180(1)
C6-C1-C2-H2	179(1)
C6-C1-C2-C3	-0.4(2)
H1-C1-C6-S1	-3(1)
H1-C1-C6-C5	179(1)
C2-C1-C6-S1	176.9(1)
C2-C1-C6-C5	-0.7(2)
C1-C2-C3-N1	-179.5(1)
C1-C2-C3-C4	1.4(2)
H2-C2-C3-N1	1(1)
H2-C2-C3-C4	-178(1)
N1-C3-C4-H4	-4(1)
N1-C3-C4-C5	179.7(1)
C2-C3-C4-H4	175(1)
C2-C3-C4-C5	-1.2(2)

C3-C4-C5-H5	179(1)
C3-C4-C5-C6	0.1(2)
H4-C4-C5-H5	3(2)
H4-C4-C5-C6	-176(1)
C4-C5-C6-S1	-176.7(1)
C4-C5-C6-C1	0.9(2)
H5-C5-C6-S1	4(1)
H5-C5-C6-C1	-178(1)
N2-C7-C8-H8	-170(1)
N2-C7-C8-C9	13.9(2)
H7A-C7-C8-H8	67(2)
H7A-C7-C8-C9	-109(1)
H7B-C7-C8-H8	-49(2)
H7B-C7-C8-C9	136(1)
C7-C8-C9-C10	-0.7(2)
C7-C8-C9-C22	173.8(1)
H8-C8-C9-C10	-176(1)
H8-C8-C9-C22	-2(1)
C8-C9-C10-H10	-179(1)
C8-C9-C10-C11	-62.0(2)
C8-C9-C10-C19	64.7(2)
C22-C9-C10-H10	6(1)
C22-C9-C10-C11	123.7(1)
C22-C9-C10-C19	-109.6(1)
C8-C9-C22-O7	32.0(2)
C8-C9-C22-O8	-146.1(1)

C10-C9-C22-O7	-153.1(1)
C10-C9-C22-O8	28.7(2)
C9-C10-C11-H11	163(1)
C9-C10-C11-C12	48.1(2)
C9-C10-C11-C13	-76.2(2)
H10-C10-C11-H11	-80(1)
H10-C10-C11-C12	165(1)
H10-C10-C11-C13	41(1)
C19-C10-C11-H11	38(1)
C19-C10-C11-C12	-76.8(1)
C19-C10-C11-C13	159.0(1)
C9-C10-C19-O5	18.1(2)
C9-C10-C19-O6	-164.4(1)
H10-C10-C19-O5	-97(1)
H10-C10-C19-O6	80(1)
C11-C10-C19-O5	145.8(1)
C11-C10-C19-O6	-36.7(2)
C10-C11-C12-N2	32.0(2)
H11-C11-C12-N2	-84(1)
C13-C11-C12-N2	160.4(1)
C10-C11-C13-C14	-134.6(1)
C10-C11-C13-C18	52.0(2)
H11-C11-C13-C14	-14(1)
H11-C11-C13-C18	172(1)
C12-C11-C13-C14	99.8(2)
C12-C11-C13-C18	-73.6(2)

C11-C13-C14-H14	7(1)
C11-C13-C14-C15	-172.4(1)
C18-C13-C14-H14	-179(1)
C18-C13-C14-C15	1.3(2)
C11-C13-C18-C17	173.0(1)
C11-C13-C18-H18	-5(1)
C14-C13-C18-C17	-0.5(2)
C14-C13-C18-H18	-179(1)
C13-C14-C15-H15	179(1)
C13-C14-C15-C16	-0.6(2)
H14-C14-C15-H15	-0(2)
H14-C14-C15-C16	-180(1)
C14-C15-C16-H16	180(1)
C14-C15-C16-C17	-0.8(2)
H15-C15-C16-H16	0(2)
H15-C15-C16-C17	179(1)
C15-C16-C17-H17	-178(1)
C15-C16-C17-C18	1.6(2)
H16-C16-C17-H17	1(2)
H16-C16-C17-C18	-179(2)
C16-C17-C18-C13	-0.9(2)
C16-C17-C18-H18	178(1)
H17-C17-C18-C13	179(1)
H17-C17-C18-H18	-3(2)
